# Promising Antidepressant Potential: The Role of *Lactobacillus rhamnosus* GG in Mental Health and Stress Response

**DOI:** 10.1007/s12602-025-10470-0

**Published:** 2025-02-17

**Authors:** Musab Işık, Fadime Köse, Cansu Özbayer, Özcan Budak, Rumeysa Keleş Kaya, Derya Güzel Erdoğan, Mehmet Arif Demirci, Songül Doğanay, Cahit Bağcı

**Affiliations:** 1 İstanbul Aydın University, Istanbul, Turkey; 2 Sakarya University, Adapazarı, Adapazarı, Turkey; 3 Kutahya Saglik Bilimleri Universitesi, Kütahya, Turkey; 4 Sağlık Bilimleri Üniversitesi, Istanbul, Turkey; 5 Muş Alparslan University, Muş, Turkey

## Abstract

Chronic stress is linked to changes in brain physiology and functioning, affects the central nervous system (CNS), and causes psychiatric diseases such as depression and anxiety. In this study, antidepressant effects of the probiotic bacterium *Lactobacillus rhamnosus* GG (ATCC 53103) (LGG) (15 × 10^8^ cfu/ml/day) on the mechanisms playing a role in the pathophysiology of depression were investigated, and the results were compared with the effects of bupropion (20 mg/kg/day) and venlafaxine (20 mg/kg/day). A total of 56 male Wistar Albino rats were used in control, stress, bupropion, venlafaxine, LGG, bupropion + stress, venlafaxine + stress, LGG + stress groups, *n* = 7 each. Changes in the body weight of the rats during the experiment were determined by weight measurement. Gene expression levels were determined by the RT-PCR method. Four different behavioral tests were performed to evaluate depressive behaviors (sucrose preference test, three-chamber sociability test (social interaction test), elevated plus maze test, forced swim test). LGG treatment was effective in reducing depressive-like behaviors, increased BDNF level, 5-HT1A, DRD1, ADRA-2A, GABA-A α1, CNR1 expression levels in the hippocampus and NOD1 receptor expression level in the small intestine (*p* < 0.05), and also decreased neurodegeneration level, glial cell activity, and intestinal permeability in depressed rats. As a result, it was revealed in this study for the first time that the LGG probiotic bacterium has antidepressant properties and was found to be more effective than the antidepressant drugs bupropion and venlafaxine. Our results suggest that LGG is a potential psychobiotic bacterium and can be useful to treat depression. It may be an effective and useful option in combating depression.

## Introduction

Stress in its current meaning, in which various defense mechanisms are activated when faced with a situation perceived as threatening or increasing demand [1], which activates biological systems that sense and respond to changes in the environment (especially situations that pose a threat to homeostasis) through neurophysiological, hormonal, and behavioral regulations [2], that is the body’s non-specific physiological response to any demand [3]. Hans Selye expressed the body’s response to stressful situations as the “General Adaptation Syndrome” and classified these stress reactions as alarm, resistance, and exhaustion stages, and defined stress as acute or chronic depending on the duration of the stressful event [4, 5]. Chronic stress persists for more than 12 months and can have negative effects on an individual’s physical, mental, and emotional health. Chronic stress can cause serious symptoms and complications such as negative changes in behavior and physiology that weaken mood, cognition, health, and longevity in the long term [6, 7]. Chronic stress is an important type of stress when it comes to mental health especially, researches show an empirical link not only to depression but also to other disorders and symptoms such as burnout and cognitive impairment. It causes psychiatric illnesses such as anxiety and depression affecting the central nervous system [8, 9].

Defined as the common cold of psychiatry, depression (major depressive disorder) is a serious and chronic brain disease that can harm mental and physical health, and is characterized by some cognitive, behavioral, and somatic symptoms [10–13]. WHO predicts that depression which is considered one of the main causes of serious economic burden in the field of health seriously affects the quality of life of individuals, and it will be the disorder with the greatest disease burden from 2030 [11, 14, 15]. Except for its chronic nature, symptoms frequently recur and are life-threatening. In the most severe case, depression can lead to suicide and is a major contributor to nearly 800,000 suicide deaths annually. The WHO reports that suicide attempts are 20 times more common than completed suicides, and depression is associated with more than 90% of all suicide cases [16–18]. A total of 10–40% of people with depression are treatment-resistant, do not respond to available medications, and these people have low quality of life and functional impairment [15, 19].

Antidepressants are the basis of treatment for depression [20]; all of them consist of drugs that affect the neurochemical balance of monoamine neurotransmitters in the CNS [21]. Although there are many drugs developed to treat depression, one of the difficulties in coping with this disease is that a significant part of patients taking antidepressants do not achieve full remission. Some patients also develop treatment-resistant depression that does not respond to available medications or other therapeutic approaches [16]. Venlafaxine, a phenylethylamine derivative that blocks the presynaptic reuptake of serotonin and norepinephrine, facilitates neurotransmission in the brain with this effect, and it is a weak inhibitor of dopamine reuptake [22]. An atypical antidepressant in many respects, bupropion is a dopamine-norepinephrine reuptake inhibitor, Wellbutrin XL tablet is available in 150 mg and 300 mg strengths and is slow-release [23, 24]. Antidepressant drugs have serious side effects such as dry mouth, sweating, tachycardia, tremor, blurred vision, sexual dysfunction, constipation, nausea, vomiting and diarrhea, sedation or insomnia, psychomotor activation, postural hypotension and dizziness, and suicide [25, 26].

In addition to the hypothalamus, the hippocampus is the first brain region acknowledged to be the target of stress and glucocorticoids and receives projections containing serotonin, norepinephrine, and dopaminergic neurons. Important projections such as cholinergic and GABAergic originate from the medial septal region and innervate all parts of the hippocampus. This projection may play a special role in maintaining the physiological state [27, 28]. The NLRP3 inflammasome plays a crucial role in stress-induced depression, suggesting that NLRP3 may represent a novel therapeutic target to cope with depressive illness [29]. Brain-derived neurotrophic factor (BDNF) plays an important role in the normal development of the CNS, increasing the survival and differentiation of neuronal populations [30]. The endocannabinoid system functions in the regulation of memory, cognition, mood, emotion, and stress responses, and it fulfills this function through the activation of the cannabinoid receptor 1 (CB1/CNR1) [31]. Melanocortin 4 receptor (MC4R) highly affects the activity of the HPA axis (hypothalamus pituitary adrenal axis) and has a functional and anatomical interaction with CRF, which is an important mediator of stress and stress-related behaviors. MC4R may be a possible therapeutic target in the treatment of stress-related disorders such as anxiety and depression [32]. Depressed patients exhibit decreased mineralocorticoid receptor (MR) expression in the hippocampus and prefrontal cortex, and several polymorphisms and haplotypes of the MR gene (NR3C2) have been associated with depression [33]. MR may be a possible target of the medicine [34]. NOD1 receptor is important for maintaining the physiological function of the gastrointestinal tract and regulates cognition, anxiety, central and peripheral serotonergic biology, and HPA axis activation. Intestinal epithelial cell expression of NOD1 receptors regulates behavior. Blocking the NOD1 receptor causes to emerge symptoms of stress-induced anxiety, cognitive impairment, and depression [35]. Stressful conditions and high corticosteroid levels are associated with accelerated damage and eventually loss of neurons [36]. Chronic stress and depression trigger apoptosis. Neuronal loss is observed in patients with depression. Activation of caspase-3 is a biomarker of neuronal apoptosis [37]. Activation of glial cells plays a role in the pathophysiology of depression [38]. A decrease in the number of glial cells results in an increase in neuron density [39]. Excessive activation of microglia plays a role in a wide variety of brain diseases including autism, neurodegenerative disorders, neuropathic pain, and depression. Microglial activation, the main mediator of neuroinflammatory processes, leads to neuroinflammation which plays a crucial role in the pathogenesis of depression. Inflammation or stress-triggered dysfunctions caused by microglia often occur in depression [40, 41]. Increased Ki-67 immunopositivity is indicative of glial activation [42]. Chronic stress increases intestinal permeability and increased intestinal permeability is seen in depression [43, 44].

According to the definition made by the Food and Agriculture Organization of the United Nations and the WHO, probiotics are live microorganisms that provide a beneficial effect on health when administered in sufficient amounts (approximately 1 × 10^9^ cfu/ml (colony forming unit)/ml cells/day) [45]. There are features that an ideal probiotic should have: it must be of human origin, safe, and have industrial properties, have a shelf life that will provide a sufficient number of live microorganisms until it is administered, able to colonize and adhere to the epithelial surface in the gastrointestinal tract, in order to ensure colonization; it must not be affected by stomach acid, duodenal secretions, and bile salts; it must be alive and in sufficient numbers in the desired region; it must temporarily colonize the intestines in order not to replace the natural intestinal flora; it must be rapidly metabolized and grow rapidly; it must have beneficial effects and it must not cause pathogenic and toxic effects in the host,; it must be able to generate a mucosal and systemic immune response and improve the immune system; it must have proteolytic activity and able to produce antimicrobial substances; and it must be resistant to antibiotics [46–50]. Probiotics have effects on the CNS and behaviors through the microbiota-gut-brain axis. Especially, there is a relationship between gut microbiota and stress response and metabolic, immune, humoral, and neural pathways may mediate the effects of probiotics on the CNS. Various probiotics, owing to their properties, regulate the HPA axis and inflammatory responses and normalize stress-induced abnormal behaviors [51]. The occurrence and development of depression is often accompanied by a decrease in Bifidobacterium and Lactobacillus species [52]. Psychobiotics are a group of probiotics that affect microbiota-gut-brain axis relationships when taken in sufficient amounts and influence CNS-related functions and behavior through metabolic, immune, humoral, and neural pathways, thus improving gastrointestinal function, antidepressant, and anxiolytic capacity. This effect of psychobiotics is mediated by the gut-brain axis. Glutamate, gamma-aminobutyric acid (GABA), serotonin, and BDNF play important roles in the control of cognitive functions, learning and memory processes, mood, and neural excitatory-inhibitory balance, and psychobiotics have the capacity to regulate these neurotransmitters and proteins and have beneficial effects on mental health in patients with psychiatric disorders [53–55].

*Lactobacillus rhamnosus* GG (LGG), isolated from healthy human gut microbiota, was discovered in 1985 as part of an attempt to isolate a Lactobacillus strain that provides the characteristics required for an ideal probiotic; it is the first strain belonging to the Lactobacillus genus and was patented in 1989 [56–58]. LGG has specific adhesive pili (fimbriae) structures, which are long and thin proteinaceous protrusions on the cell surface found in specific gram-positive bacterium, and therefore adheres effectively to the gastrointestinal mucosa in both adults and children. It can form biofilms on abiotic surfaces and cells exposed to 8000 g centrifugal force lose their structure. LGG is resistant to gastric acid and bile and has good growth properties and the capacity to adhere to the intestinal epithelial layer [57, 59–61]. LGG is one of the probiotic strains that is best clinically studied, most widely used, and has rapid growth properties. After oral administration, its duration in the intestines is more than a week and it can also colonize in the mouth [56, 61]. LGG, a bacterium that exists in the gastrointestinal tract and maintains intestinal homeostasis, is effective in antibacterial compound production, regulation of the immune response and intestinal flora, and neurotransmitter modulation [62]. Numerous research data on LGG form the basis for the consumption of this probiotic for human health [58]. LGG is a bacterium that positively affects the gut-brain axis by reducing bacterial translocation in the intestine, restoring intestinal and blood–brain barrier functions, and improving intestinal bacterial balance [63]. LGG is an effective bacterium in increasing the levels of neurotransmitters that play important roles in depression, such as serotonin, noradrenaline, dopamine, and GABA [64]. LGG has an effect of reducing neuroinflammation that leads to the death of neurons [65]. The capacity of LGG to adhere to the intestinal epithelium is superior to that of other Lactobacillus strains and it supports the vitality of intestinal cells more than other probiotic Lactobacillus strains [66]. Our previous study has shown that LGG at a dose of 15 × 10^8^ cfu/ml prevents excessive activation of the hypothalamic–pituitary–adrenal axis and has antistress properties [67].

Since antidepressants used today have serious side effects and do not provide the desired level of treatment response, there is a need for alternative methods that are safe in terms of side effects and effective in terms of therapeutic properties. In this study, we aimed to investigate the effects of *Lactobacillus rhamnosus* GG probiotic bacterium on the mechanisms playing a role in the pathophysiology of chronic stress-induced depression. Our study is the first comprehensive study in the literature to examine the effects of LGG strain on depression in a chronic stress model at behavioral, histological, and molecular levels. There is no study in the literature investigating the effects of LGG on mood changes and its effects on the cannabinoid system. It also reveals the analgesic and sedative effects of LGG via the hippocampus for the first time.

## Methods

### Experimental Animals and Housing

In this study, 56 rats randomly selected among healthy 2-month-old, about 200 g Wistar Albino males were used in 8 groups, each *n* = 7. Rats were purchased from Sakarya University Faculty of Medicine Experimental Medical Applications and Research Center, and the experiment was performed in this center. The rats placed in the new living environment where the treatments would be administered waited for 1 week to adapt. Rats in groups exposed to stress were housed in a separate and distant room from other groups. During the applications, all experimental animals were kept in rooms with 12/12 light/dark lighting, temperature (22 ± 2 °C), and humidity (45–50%) optimized, in polycarbonate transparent cages, fed with standard pellet feed and provided tap water.

### Preparation of LGG

*Lactobacillus rhamnosus* GG (ATCC 53103) was provided from CHR HANSEN A/S. MRS Broth liquid medium (Merck) was prepared fresh in the desired amount based on 52.2 g/L, placed in test tubes, and sterilized with dry heat in the oven at 121 °C for 15 min. Then the medium was allowed to cool. After cooling, LGG was inoculated in MRS Broth liquid medium. It was incubated at 37 °C for 24 h. At the end of 24 h, the test tubes containing the bacterium were centrifuged at 2500 rpm for 3 min to ensure that the bacterium precipitated. The liquid part, the medium, was removed. Thus, it was ensured that only bacterium remained in the test tubes. The bacterium was washed twice with sterile PBS, and the medium was completely removed. Sterile water for injection was added to the test tubes, and the bacterial content was prepared according to the McFarland-5 standard (15 × 10^8^ cfu/ml), whose density was determined on the McFarland unit cell densitometer, and using a Wickerham card. In order for the bacterium to show the highest activity, bacterium was incubated every day and fresh bacterium was administered to the experimental animals by gavage [68, 69].

### Preparation of Venlafaxine HCl Content

Venlafaxine HCl was provided from İLKO Pharmaceuticals. Venlafaxine at a dose of 20 mg/kg has been shown to provide an antidepressant effect in reducing the effects of chronic stress, and therefore, this dose was chosen in our study [70]. Thirty-two milligrams of venlafaxine HCl as a lyophilized powder pure substance was dissolved in 8 ml sterile water for injection and 4 mg/ml venlafaxine HCl content was obtained. Venlafaxine HCl content was prepared fresh every day and administered to the experimental animals by gavage.

### Preparation of Bupropion (WELLBUTRIN XL 150 mg) Content

Bupropion (WELLBUTRIN XL 150 mg) was provided from the pharmacy (Pelin Pharmacy). A dose of 20 mg/kg bupropion is effective in antidepressant effects, and therefore, this dose was chosen in our study [71]. One hundred fifty milligrams of Bupropion tablet was dissolved in 37.5 ml sterile water for injection, and 4 mg/ml bupropion content was obtained. Bupropion content was prepared fresh every day and administered to experimental animals by gavage.

### Experimental Design

**Fig. 1:**
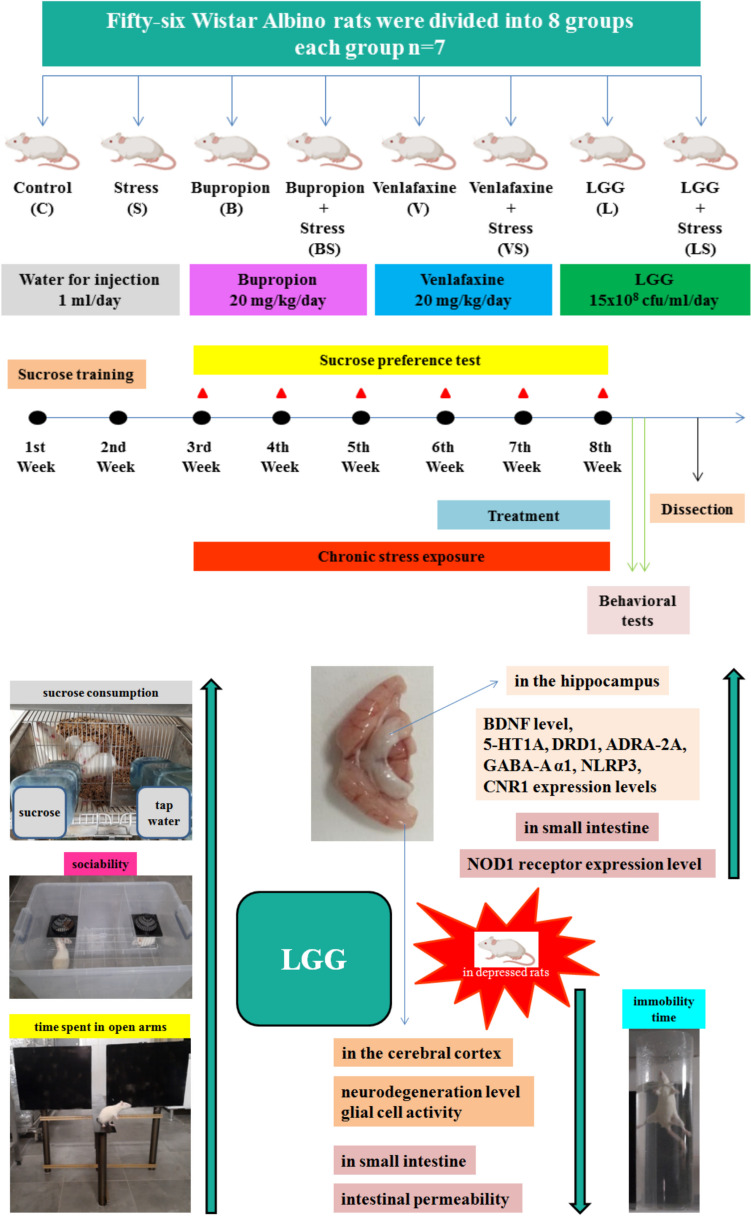
Schematic representation of the experimental design and antidepressant effects of LGG in the depressed rats

The procedures applied to the experimental groups are shown in Table 1. Rats in the S, BS, VS, and LS groups were exposed to chronic stress. The experimental design is shown in Fig. 1.

**Table 1:** Experimental groups

Groups	Administered substance	Dose	Volume	Method	Duration
Control (C)	Water for injection	1 ml/day	1 ml	Gavage	21 days
Stress (S)	Water for injection	1 ml/day	1 ml	Gavage	21 days
Bupropion (B)	Bupropion	20 mg/kg/day	1 ml	Gavage	21 days
Bupropion + stress (BS)	Bupropion	20 mg/kg/day	1 ml	Gavage	21 days
Venlafaxine (V)	Venlafaxine	20 mg/kg/day	1 ml	Gavage	21 days
Venlafaxine + stress (VS)	Venlafaxine	20 mg/kg/day	1 ml	Gavage	21 days
LGG (L)	LGG	15 × 10^8^ cfu/ml/day	1 ml	Gavage	21 days
LGG + stress (LS)	LGG	15 × 10^8^ cfu/ml/day	1 ml	Gavage	21 days

#### Chronic Unpredictable Mild Stress (CUMS) Procedure

The CUMS model is widely recognized as the most reliable and effective method for inducing depression in rodents. The CUMS protocol serves as a dependable method for simulating depression. This model prevents adaptation to repeated stress by presenting a range of stressors in a random and unpredictable sequence. Chronic unpredictable mild stress (CUMS) procedure performed according to the Willner protocol (modified) [72–76]; the stressors included in the chronic stress protocol and applied to rats are shown in Table 2. The chronic stress protocol continued for 8 weeks. The stressors were applied to rats in stress groups on different days each week to prevent adaptation of rats to the stressors. It was determined according to the results of the sucrose preference test performed starting from the 3rd week to determine if the rats in the stress groups were stressed. Considering the results obtained, depression treatment was started in the 6th week.

**Table 2:** Chronic stress protocol

Stressor	Duration
45° cage tilt	Twice a week, 21–24 h
Wet sawdust	Once a week, 21 h
Leaving alone in dark cage 12 × 12 × 12 cm^3^ sized (daytime)	Once a week, 1–2 h
Immobilization at + 4 °C	Once a week, 3 h
Changing the cage (coming together with stranger partners)	Once a week, 2–3 h
Food and water deprivation	Once a week, 21–24 h
Restricted access to food (5 pellets)	Once a week, 1 h
Overnight illumination	Twice a week
Loud noise (85–90 dB)	3 times a week, 3 h
Bright flashing light (300 times/min)	3 times a week, 3 h

#### Body Weight Measurement (g)

At the end of the second, fifth, and eighth (14th, 35th, and 56th days) weeks, the body weights of the rats (as group) were measured using an electronic scale.

#### Behavioral Tests

The sucrose preference test was performed once a week starting from the 3rd week, and other tests were performed at the end of the experiment (at the end of the 8th week). Behavioral tests were performed as elevated plus maze test, three-chamber sociability test, and forced swim test (FST) in order not to increase the stress levels of the animals and to ensure the reliability of the results. There is no acclimatization period before the tests except the forced swim test.

##### Sucrose Preference Test (SPT)

**Fig. 2:**
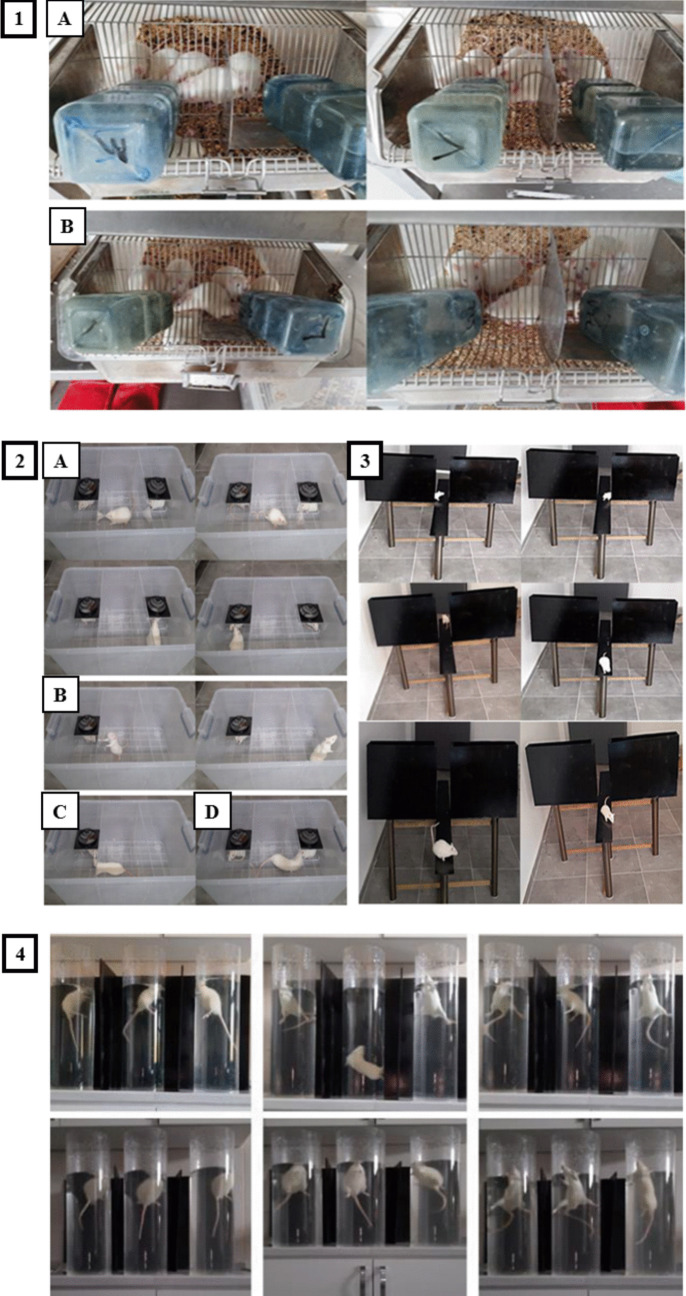
1) Sucrose preference test (SPT):** A**) Rats that prefer sucrose water **B**) Rats that prefer both sucrose water and tap water, 2) **A**) Three-chamber sociability test; **B**) Rearing **C**) Social interaction with one stranger partner **D**) Social interaction with second stranger partner, 3) Elevated plus maze test 4) Forced swim test (FST)

SPT was used to determine anhedonia, defined as a decrease in sucrose preference relative to basal levels. Rats prefer sucrose water to tap water, and if sucrose water preference is below 65%, rats are considered to show anhedonia. When rats are not interested in sucrose water, it is accepted to exhibit anhedonia, a loss of interest in pleasurable things, which is a classic symptom of depression. To determine sucrose preference for 6 weeks, two 200-ml water bottles, one containing tap water and the other containing 1% sucrose solution, were placed in cages for 1 h for rats deprived of food and tap water for 24 h. At the end of 1 h, sucrose consumption was calculated according to the formula: Sucrose preference = sucrose consumption/(sucrose consumption + tap water consumption) × 100 [77–80], (Fig. 2. 1(A), (B)). Before starting the sucrose preference test, all experimental animals were subjected to sucrose water training for 2 weeks. Two bottles containing 500 ml of 1% sucrose water were prepared. On the 1st day, 2 bottles containing sucrose water were placed in the cages. On the 2nd day, a sucrose water bottle was replaced with a bottle containing 500 ml tap water. On the 3rd day, rats were deprived of food and water for 24 h. On the 4th day, 1 sucrose water bottle and 1 tap water bottle were placed in the cages, and sucrose consumption was calculated at the end of the 1-h period [81].

##### Elevated Plus Maze Test

Anxiety-like behaviors are tested with a plus-shaped device having two open (50 × 10 cm) and two closed (50 × 10 × 40 cm) arms, with a 10 × 10 cm area connecting these arms in the center, usually 50–70 cm high from the ground. Rats tend to avoid luminous places, but they also tend to discovery new areas. This test is the most preferred anxiety test, based on the conflict between te urge to discovery a new environment and the fear of open and high spaces of rats. Depending on the decrease in anxiety, the time spent in open arms and the number of enter to open arms increase. The elevated plus maze test is often used in conjunction with the forced swim test, which is used in studies of depression. The dark black device used for the test was made of glass + metal mixture material, consisting of two open arms of 10 × 110 cm sized and two closed arms of 10 × 40 × 50 cm sized, with a height of 50 cm from the ground [77, 81–84]. Experimental animals were placed in the center of the plus maze-shaped device, facing the open arm and the time spent in the open arms and closed arms was recorded for 5 min. Open and closed arms were cleaned with 10% alcohol and dried after each rat was tested (Fig. 2 3).

##### Three-Chamber Sociability Test (Social İnteraction Test)

It is an anxiety model (test) that analyzes social behavioral reactions. The social interaction test, which is sensitive to a range of environmental and physiological factors that may influence anxiety, provides an ethologically based test that is sensitive to both anxiolytic and anxiogenic effects. The dependent variable is the time that two stranger male rats spend in social interaction (following, sniffing, or grooming the partner). An anxiolytic effect is an increase in social interaction without a contemporaneous increase in motor activity; the anxiogenic effect is a specific reduction in social interaction. Test conditions can be modulated to produce different levels of anxiety; both the light brightness and the test place can be changed. Therefore, there are four testing conditions: dim light, familiar place (producing the lowest level of anxiety); bright light, familiar place and dim light, unfamiliar place (produces moderate anxiety); bright light, unfamiliar place (produces highest levels of anxiety). Social interaction is highest level when rats are tested in a familiar place with dim light and social interaction decreases as testing conditions become more aversive or anxiety-provoking [85–87]. In the present study dim light, unfamiliar place that produced moderate levels of anxiety was chosen as the test condition. For the test, a cage made of transparent resistant plastic material 40 × 35 × 60 cm sized and divided into three equal chambers (40 × 35 × 20 cm sized) with two transparent plexiglass plates 40 × 35 cm sized was used. In order for the rats to pass between the chambers, 5 × 5 cm-sized passage holes were made in the middle of the bottom of the plexiglass plates [88–91]. Each rat was placed in the middle chamber firstly, and at the same time, a stranger male rat in a small cage was placed in the left chamber of the test cage. After 150 s, a second stranger male rat in a small cage was placed in the right chamber. The interactions, rearing numbers, and the time spent of each experimental animal in three different chambers in the cage were recorded for 5 min. The small cages were designed to allow nose-to-nose contact but to prevent possible fighting between the rats. The duration of snuggling, following, climbing on, and eye tracking each other and the number of rearing were recorded as social interaction time (Fig. 2 2 (A), (B), (C), (D)).

##### Forced Swim Test (FST)

The forced swim test (also known as the Porsolt test; behavioral despair test) is the most common and often used experimental model to determine clinical antidepressant activity. Rats develop a motionless posture following initial escape movements in a water-filled cylinder. If antidepressant treatments are administered before the test, rats actively continue to maintain escape-oriented behaviors for longer periods of time. For the test, 20 × 60 cm sized three cylindrical water tanks made of transparent plexiglass material were used. The test consists of two sessions [83, 92–94]. In both sessions, clean water at a temperature of 24–26 °C was used, and when the water became soiled, it was replaced with clean water. The first session, which lasted 15 min was done to acclimate the rats to the test environment. This session was performed before treatment was administered and without behavioral recording. In the second session performed 24 h after the first session and lasted 5 min, the periods which the rats remained completely motionless were recorded. After 5 min, the rats were dried with a towel and then dried entirely using a hair dryer. Additionally, a heater was placed next to the cage to prevent the rats from getting cold (Fig. 2 4).

#### Collecting Tissue Samples

After the behavioral tests performed at the end of the 8th week, the rats were sacrificed under ketamine (80 mg/kg)-xylazine (5 mg/kg) anesthesia and the tissues (hippocampus, cortex, ileum part of the small intestine) were collected and stored at − 80 °C until examined. Also, cortex and ileum tissues placed in histology cassettes were put in a 10% buffered neutral formaldehyde solution for histopathological examinations.

#### Determination of BDNF Level

BDNF levels in hippocampus tissue were determined using the ELK Biotechnology Rat BDNF Elisa Kit (ELK5459) in accordance with the manufacturer’s instructions.

#### Determination of 5-HT1A, DRD1, ADRA-2A, GABA-Α α1, CNR1, MC4R, NR3C2, and NLRP3 in Hippocampus Tissue and NOD1 Receptor Expression Levels in Small Intestine Tissue by RT-PCR

The expression level of the genes planned to be studied in the hippocampus and small intestine tissues was determined using the Promega A6001 RT PCR kit, in accordance with the manufacturer’s instructions. Primers are shown in Table 3.

**Table 3:** Primers

Gene	Prımer	Sequence
5-HT1A (388 bp)	Forward	5′-CCAAAGAGCACCTTCCTCTG-3′
	Reverse	5′-TACCACCACCATCATCATCA-3′
DRD1 (108 bp)	Forward	5′-GACACAAGGTTGAGCA-3′
	Reverse	5′-CTGGGCAATCCTGTAGATA-3′
ADRA2a (112 bp)	Forward	5′-TTCTTTTTCACCTACACGCTCA-3′
	Reverse	5′-TGTAGATAACAGGGTTCAGCGA-3′
GABA-A α1 (304 bp)	Forward	5′-AGCTATACCCCTAACTTAGCCAGG-3′
	Reverse	5′-AGAAAGCGATTCTCAGTGCAGAGG-3′
CNR1 (306 bp)	Forward	5′-ATGAAGTCGATCCTAGATGGCCTTG-3′
	Reverse	5′-GTTCTCCCCACACTGGATG-3′
MC4R (431 bp)	Forward	5′-AGTCTCTGGGGAAGGGGCA-3′
	Reverse	5′-CAACTGATGATGATCCCGAC-3′
NR3C2 (260 bp)	Forward	5′-GCTCAACATTGTCCAGTACA-3′
	Reverse	5′-GCACAGGTGGTCCTAAGATT-3′
NLRP3 (313 bp)	Forward	5′-TCTGTTCATTGGCTGCGGAT-3′
	Reverse	5′-GCCTTTTTCGAACTTGCCGT-3′
NOD1 (149 bp)	Forward	5′-TAGCCTTCTGCAATGCTTGTTC-3′
	Reverse	5′-CCGTGAGACGGCTAAAGCAA-3′
β-actin (150 bp)	Forward	5′-CCTGTGGCATCCATGAAACTAC-3′
	Reverse	5′-CCAGGGCAGTAATCTCCTTCTG-3′

#### Histopathological Analysis in The Cortex and Small Intestine Tissues

All tissue samples taken were placed in 10% buffered neutral formaldehyde solution (formaldehyde 100 cc; tap water 900 cc; NaH2PO4.H2O4 g; Na2HP046.5 g) for light microscopic examination and kept for 48 h for tissue fixation. Tissue samples fixed with buffered neutral formaldehyde were washed in tap water following the trimming process at the end of 48 h and then put tissue through processing steps in the MTP semi-closed system automatic tissue processing machine. Tissue samples for which fixation procedures were completed were embedded in paraffin. Sections of 4 µm thickness, taken from the prepared paraffin blocks with a microtome, were placed on slides for H-E staining and kept at room temperature to dry until the staining process. After the H-E staining process was completed, the slides were covered with entellan and made ready to be examined under the microscope.

##### Histopathological Evaluation

Damaged neurons in the cerebral cortex after chronic stress exposure were examined in the prepared preparations. When evaluating the effects of chronic stress histologically, we based on the presence and number of red neurons defined by acidophilic neuronal cytoplasm, pyknotic nuclei, and karyorrhexis. Damaged neurons have acidophilic cytoplasm and are called red neurons. However, spongy decomposition areas in the neuropil formed by neuroglial cells clustered around damaged neurons are called satellitosis and spongiosis. While neuronal damage was evaluated under the light microscope, damaged neurons were counted in 5 different areas at 20X magnification. To determine changes in intestinal permeability in rats in the control and stress groups, villus structures, submucosa and muscularis layers, and ulceration areas were examined [95–97].

##### Immunohistochemistry Staining Protocol

Caspase-3 antibodies (Caspase-3 Antibody Brand: Genetex) were used to show the number of damaged cells in the tissue, and Ki-67 antibodies (Ki-67 Antibody Brand: Genetex) were used to show cell proliferation. Caspase-3 and Ki-67 staining percentages were determined by the H-Score method. The degree of staining was scored in five randomly selected areas, and the area with the highest score was determined. For both groups, at least 200 cells were labeled in each at 40X magnification areas. In the sections, the percentage of stained cells and the degree of staining were taken as criteria. The scoring was done with a semiquantitative method. The degree of staining was evaluated as 0 (no staining), + 1 (weak staining), + 2 (moderate staining), and + 3 (strong staining). H Score = (3 × percentage of strongly staining nucleus) + (2 × percentage of moderately staining nucleus) + (1 × percentage of weakly staining nucleus) [98].

#### Statistical Analysis

Analyzes were made using the GraphPad Prism 8 program. Parametric tests are used in cases where the data set exhibits a normal distribution. Parametric tests are more powerful tests than non-parametric tests. The suitability of the groups for normal distribution was determined by the Shapiro–Wilk test, the degree of proximity of the mean and median values, and the skewness and kurtosis criteria. Because the groups exhibited normal distribution, one of the parametric tests, one-way analysis of variance (one-way ANOVA) was used for comparison between groups. In multiple comparisons, Dunnet’s test was used to compare the control group with other groups while Tukey’s HSD test was used to compare other groups except the control group. By taking the statistical significance level as 0.05, it was decided with 95% confidence whether the differences between the means were statistically significant. The statistical significance of the data in the present study was expressed as *p < 0.05, **p < 0.01 and ***p < 0.001.

## Results

### Effects of Bupropion, Venlafaxine, and LGG on the Body Weight

**Fig. 3:**
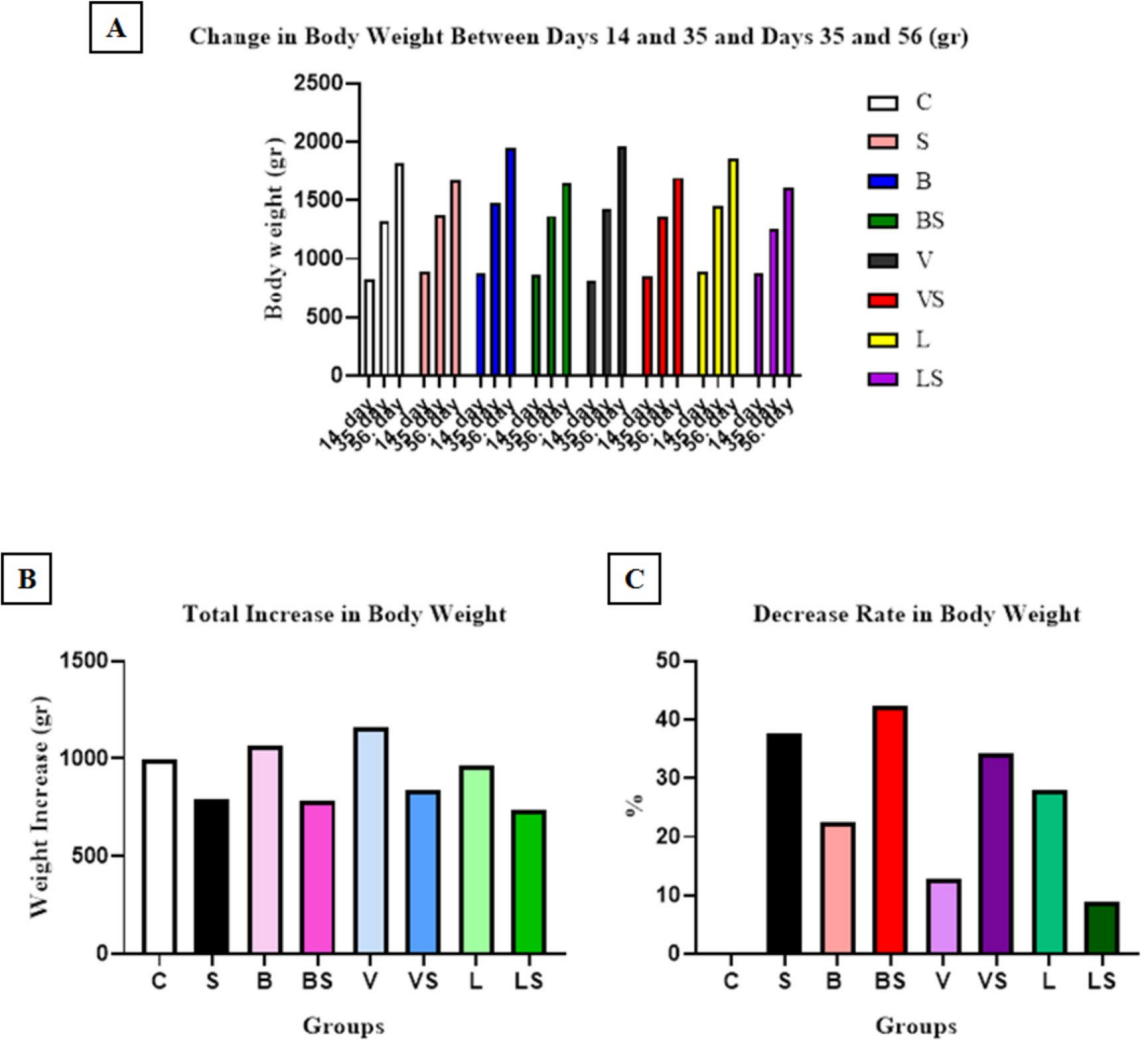
**A** Change in body weight (g) on the 14th, 35th and 56th days. **B** Total body weight gain (g) of the experimental groups during the experiment **C**) Decrease rate in body weight gain between 14-35th and 35-56th days

To determine the effect of the change in appetite, which is one of the symptoms of depression, the body weights of the rats were measured separately as a group at the end of the 2nd, 5th, and 8th weeks. According to the results obtained in the study, total body weight change was found at similar levels in the stressed groups, and the body weight increase was less than in the C group. While a higher body weight increase was observed in groups B and V compared to the C group, a similar body weight increase was observed in the C and L groups. According to the measurements made on the 14th, 35th, and 56th days, differences were observed in the body weights of all experimental groups. There was no decrease in body weight increase in the C group, the most decrease in body weight between the 14th and 35th days and the 35th and 56th days was seen respectively in the BS, S, and VS groups due to the effects of chronic stress (Fig 3).

### Effects of Bupropion, Venlafaxine, and LGG on the Behavioral Tests

**Fig. 4:**
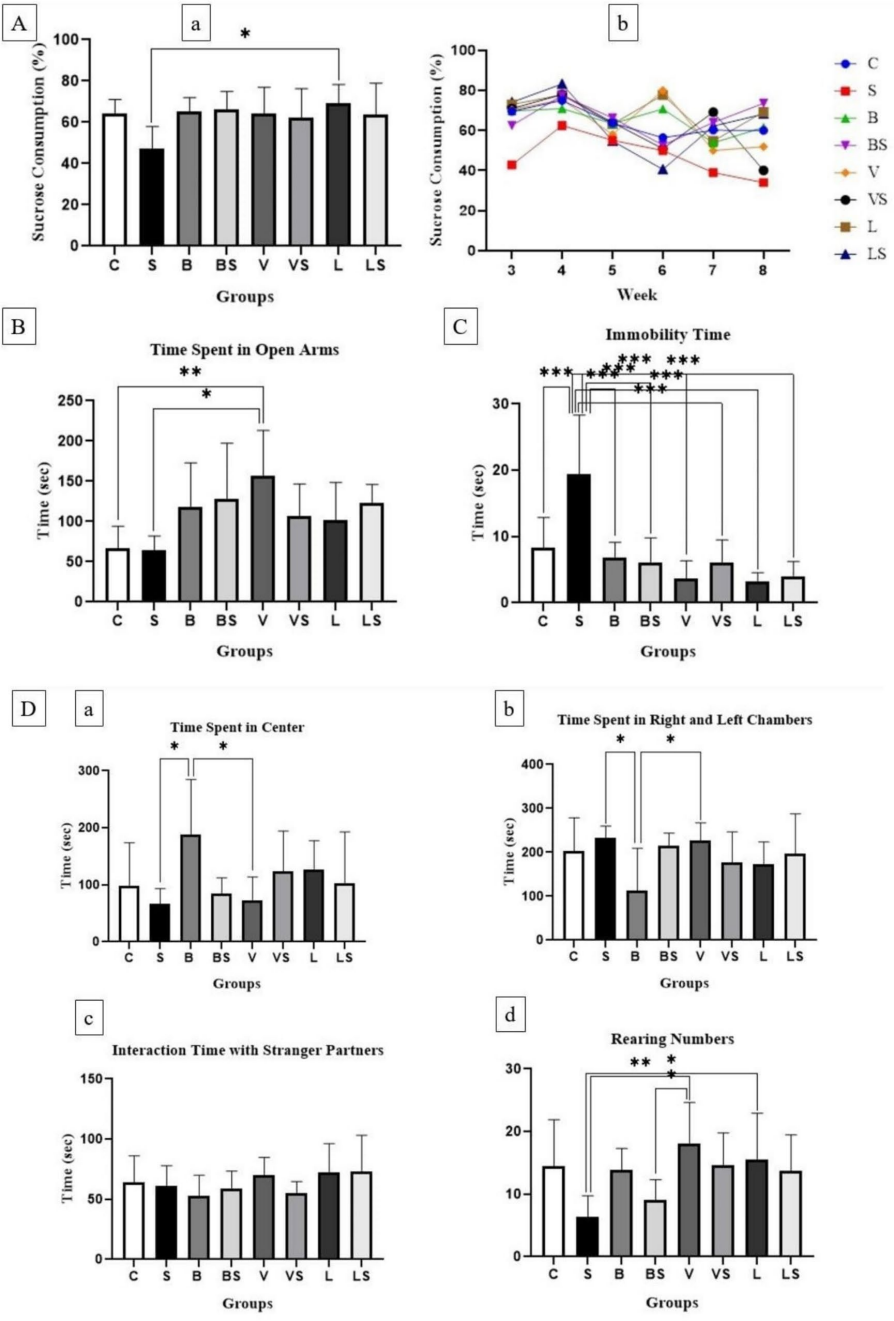
**A** Sucrose preference test: (a) Percentage of sucrose consumption rate (%) and (b) weekly change in sucrose consumption (%), (F:2,21; *p*>0,05) **B**) Elevated plus maze test: Time spent in open arms (F:3,37; **p<0,01) **C**) Forced swim test (FST): Immobility time (F:10,67; ***p<0,001) **D**) Three-chamber sociability test (social interaction test): (a) time spent in center (F:2,48; *p<0,05), (b) time spent in right and left chambers (F:2,48; *p<0,05), (c) interaction time with stranger partners (F:1,14; *p*>0,05), (d) rearing numbers (F:3,17; **p<0,01)

Sucrose Preference Test Results: In our study, when the 6-week total sucrose consumption was compared between the groups, a chronic stress-induced decrease in sucrose consumption was observed in the S group, while an increase in sucrose consumption was observed in the other stressed groups due to the effect of the treatments. Sucrose consumption decreased in the S group compared to the C group, and the highest sucrose consumption was observed in the L group. Similar rates of sucrose consumption were observed in the B, BS, V, and LS groups. There was no statistically significant difference between the groups (F:2.21; *p* > 0.05). Sucrose consumption tended to decrease continuously in the S group starting from the 4th week. Fluctuations were observed in the C, B, V, and L groups over the 6-week period. An increase in sucrose consumption was seen in the BS and LS groups a week after bupropion and *Lactobacillus rhamnosus* GG treatment started. While there was an increase in sucrose consumption in the VS group 1 week after venlafaxine treatment started, a decrease was seen in the last week (Fig. 4.A (a), (b)).

Elevated Plus Maze Test Results: In our study, rats in the S group spent the least time in the open arms, and the time spent in the open arms of the S and C groups was close to each other. Rats in all other experimental groups spent time in the open arms for longer periods of time than the C and S groups. Rats in the V group spent time in the open arms for the longest time; however, the time spent in the open arm decreased in the VS group compared to the V group and this shows that the effect of venlafaxine decreases due to stress. An increase was observed in the time spent in the open arms of the BS group compared to the B group and this shows that bupropion is effective in stressful situations. Rats in the BS and LS groups spent time in the open arms for similar periods of time. LGG was more effective in stressful condition than in the non-stressful condition. A statistically significant difference was found between the C-V and S-V groups (F:3.37; **p < 0.01). In the plus maze test, venlafaxine, bupropion, and LGG supplement were effective in the stressed groups compared to the untreated group, but this effect was prominently in the groups treated with bupropion and LGG. The results we found are compatible with the results of studies in the literature (Fig. 4B).

Forced Swim Test Results: In our study, the rats in the S group remained motionless for a longer time than the rats in the C group. The immobility time of rats in the B, BS, and VS groups was similar to the C group. The shortest immobility time was observed in the L, LS, and VS groups and the immobility time of the rats in these three groups was shorter than the C group. A statistically significant difference was found between the C-S, S-B, S-BS, S-V, S-VS, S-L, and S-LS groups (F:10.67; ***p < 0.001). Our results show that bupropion, venlafaxine, and LGG probiotic bacteria are effective in reversing depressive behaviors caused by chronic stress and are compatible with the results of studies in the literature (Fig. 2C).

Three-Chamber Sociability Test Results: In our study, the time experimental animals spent in the center differed between groups. B group spent the most time in the center. VS and L groups, which spent time in the center for a bit more long time than the C group, stayed in the center for similar periods of time. While C and LS groups stayed in the center for a similar period of time, S, V, and BS groups respectively were the groups that spent the shortest period of time in the center. A statistically significant difference was found between the S-B and B-V groups (F:2.48; *p < 0.05). While the time spent in the right and left chambers was more in the S and V groups than in the C group, it was observed shorter in the B, L, and VS groups. The time spent in the right and left chambers in the LS and BS groups was similar to the C group. Bupropion treatment reduced the time spent in the center, while LGG had a similar effect to the control group. Bupropion also increased time spent in the right and left chambers. A statistically significant difference was found between the S-B and B-V groups (F:2.48; *p < 0.05). Compared to the C group, rats in the LS, L, and V groups spent more time with stranger partners. LGG was effective in increasing interaction time. Rats in the other groups interacted with stranger partners for less time than the C group. Rats in the B and VS groups interacted with stranger partners for less time compared to the S group. There was no statistically significant difference between the groups (F:1.14; *p* > 0.05). Very few rearings were seen in the S group compared to the C group, and a similar number of rearings were seen in the BS group compared to the S group. The number of rearings was similar in the B, VS, LS, and C groups. The most numerous rearings were observed in the V group. The more number of rearings, which are important for curiosity and getting to know the new environment, was observed in the stress + treatment groups compared to the S group. A statistically significant difference was found between the S-V, S-L, and BS-V groups (F:3.17; **p < 0.01) (Fig. 4D (a), (b), (c), (d)).

### Spectrophotometric Analyses of BDNF and Evaluation of Gene Expression Results Determined by RT-PCR Method

**Fig. 5:**
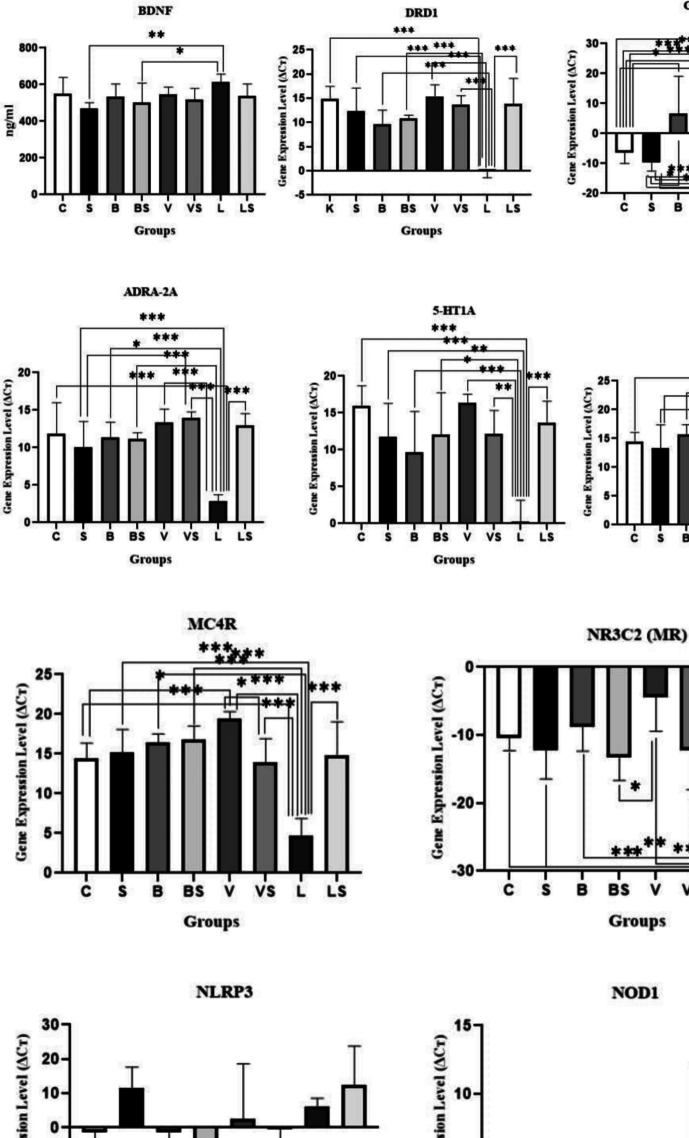
BDNF level (F:2,65; *p<0,05) and DRD1 (F:13,12; ***p<0,001), CNR1 (F:12,21; ***p<0,001), ADRA-2A (F:12,11; ***p<0,001), 5-HT1A (F:8,55; ***p<0,001), GABA-Α α1 (F:11,58; ***p<0,001), MC4R (F:15,59; ***p<0,001), NR3C2 (F:6,75; ***p<0,001), NLRP3 (F:1,88; *p*>0,05) in hippocampus tissue and NOD1 receptor expression levels in small intestine tissue

BDNF Level: According to our results, BDNF level decreased in the S group compared to the C group and it was found slightly higher in the BS, V, and LS groups compared to the S group. While the BDNF level in the B and VS groups was similar to the C group, the highest BDNF level was seen in the L group. A statistically significant difference was found between the S-L and BS-L groups (F:2.65; *p < 0.05), (Fig. 5).

#### Gene Expression Results

While there was a decrease in DRD1 expression level in the S group compared to the C group, a decrease was observed in the B and BS groups compared to the S group. The expression level was observed at a similar level in the VS, LS, and C groups. It was downregulated in the L group. A statistically significant difference was found between the C-L, S-L, B-L, BS-L, V-L, VS-L, and L-LS groups (F:13.12; ***p < 0.001), (Fig. 5).

We found that 5-HT1A receptor expression was reduced in the hippocampus of rats in group S compared to group C, the expression level in the BS and VS groups was found close to the S group. 5-HT1A receptor expression was seen at a very low level in the L group. It was observed that the expression level increased in the LS group which decreased with the effect of chronic stress. While the highest expression level was seen in the V group, a decrease was seen in the B group compared to the S group. A statistically significant difference was found between the C-L, S-L, B-L, BS-L, V-L, VS-L, and L-LS groups (F:8.55; ***p < 0.001), (Fig. 5).

The ADRA-2A receptor expression level decreased in the S group compared to the C group; its expression level increased in the B and BS groups compared to the S group. The expression level was found higher in the V, VS, and LS groups than in the C group. ADRA-2A receptor expression was observed at low levels in the L group. A statistically significant difference was found between the C-L, S-L, B-L, BS-L, V-L, VS-L, and L-LS groups (F:12.11; ***p < 0.001), (Fig. 5).

According to the results of our study, the GABA-A α1 receptor expression level decreased in the S group compared to the C group; however, there was not much difference in the expression level, and a decreased expression level was observed in the L group compared to the S group. While there was a slight increase in the BS group compared to the S group, the expression level was similar to each other in the B, VS, and LS groups and was higher than the C group. The highest GABA-A α1 receptor expression level was found in the V group. A statistically significant difference was found between the C-L, S-V, S-L, B-L, BS-L, V-L, VS-L, and L-LS groups (F:11.58; ***p < 0.001), (Fig. 5). The expression level decreased in the BS and VS groups compared to the B and V groups respectively but the expression level increased in the LS group compared to the L group.

We found in our study that the CNR1 expression level was downregulated in the C and S groups, the downregulation level was more in the S group, and the expression level was found upregulated in the other groups. While similar results were seen in the B-L and BS-V groups, the highest CNR1 expression level was seen in the LS group and then in the VS and BS groups, respectively. A statistically significant difference was found between the K-B, BS, V, VS, L, LS and S-B, BS, V, VS, L, LS groups (F:12.21; ***p < 0.001), (Fig. 3).

In the current study, while there was a slight increase in the S group compared to the C group, higher MC4R expression level was found in the B, BS, and V groups than in the S group. Similar results were seen in the LS and C groups, and a decreased expression level was found in the VS group compared to the S and C groups. The lowest MC4R expression level was seen in the L group. A statistically significant difference was found between the C-L, C-V, S-L, B-L, BS-L, V-L, VS-L, V-VS, and L-LS groups (F:15.59; ***p < 0.001), (Fig. 5). Venlafaxine was effective in decreasing chronic stress-induced increased MC4R expression.

NR3C2 receptor expression level was downregulated in all experimental groups. The level of downregulation was different between the groups. In stressed groups, the lowest downregulation level was seen in the LS group. A statistically significant difference was found between the C-L, C-V, S-L, B-L, BS-L, V-L, VS-L, V-VS, and L-LS groups (F:6.75; ***p < 0.001), (Fig. 5).

The expression level of NLRP3 is downregulated in the C, B, and BS groups. While there was an increased expression level in the S and LS groups compared to the C group, a less increased expression level was found in the V, L, and VS groups compared to the C group. No statistically significant difference was found between groups (F:1.88; *p* > 0.05), (Fig. 5).

NOD1 receptor expression level decreased in the S group compared to the C group and decreased expression level was observed in the V and VS groups compared to the S group. While the B, BS, and LS groups had results similar to the C group, the highest expression level was found in the L group. A statistically significant difference was found between VS-L groups (F:2.39; *p < 0.05), (Fig. 5).

### Histopathological Analysis in the Cortex and Small Intestine Tissues

**Fig. 6:**
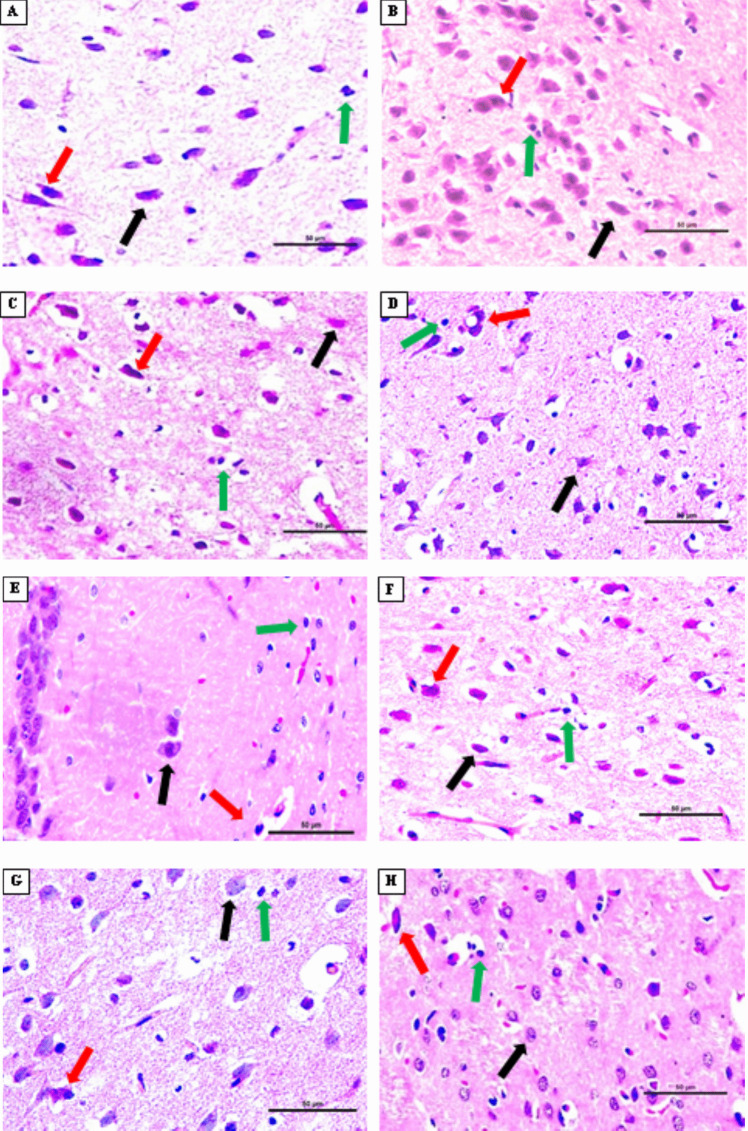
Histological evaluation of the cortex tissue: red neurons and neuronal satellitosis. Control group (**A**), stress group (**B**), bupropion group (**C**), bupropion + stress group (**D**), venlafaxine group (**E**), venlafaxine + stress group (**F**), LGG group (**G**), LGG + stress group (**H**), (bar: 50.0μm, 20X, HE). Black arrows show normal neurons, red arrows show damaged neurons, and green arrows show increased number of neuroglial cells (neuronal satellitosis)

Red neurons indicate the degree of neuronal damage, that is, neurodegeneration, and in the current study, the number of red neurons in the V, L, and LS groups was similar to the C group; a slightly greater number of red neurons were observed in the B group than the C group. Greater red neurons were observed in the VS and BS groups than in the C group. The greatest number of red neurons was observed in the S group. Among the chronic stress groups treated, the LS group had the lowest number of red neurons. Caspase-3 was used to show the number of neurons undergoing apoptosis in the cortex tissue. The number of neurons undergoing apoptosis in groups V and L was similar to the C group, but the number of neurons undergoing apoptosis was slightly greater in the B group than in the C group. Greater apoptotic neuron cells were seen in the LS, VS, and BS groups, respectively, compared to the C group. The greatest numbers of apoptotic neuron cells were seen in the S group (Figures 6, 8 1, 3).

Satellitosis is the accumulation of great numbers of oligodendroglia or microglia around damaged neuron cells, and in our study, the number of neuronal satellitosis in the V, L, and LS groups was similar to the C group; it was slightly greater in the B group than in the C group. A greater number of neuronal satellitosis was observed in the VS and BS groups compared to the C group. The lowest number of neuronal satellitosis was found in the LS group, and the greatest number of neuronal satellitosis was observed in the S group. Ki-67 was used to demonstrate cell proliferation, that is, glial activation in the cortex tissue. The glial activation level in the B, V, and L groups was found similar to the C group. While a slightly increased glial activation level was found in the LS group compared to the C group, an increased glial activation level was found in the BS and VS groups compared to the LS group. The highest level of glial activation was found in the S group (Figures 6, 8 2, 3).

**Fig. 7:**
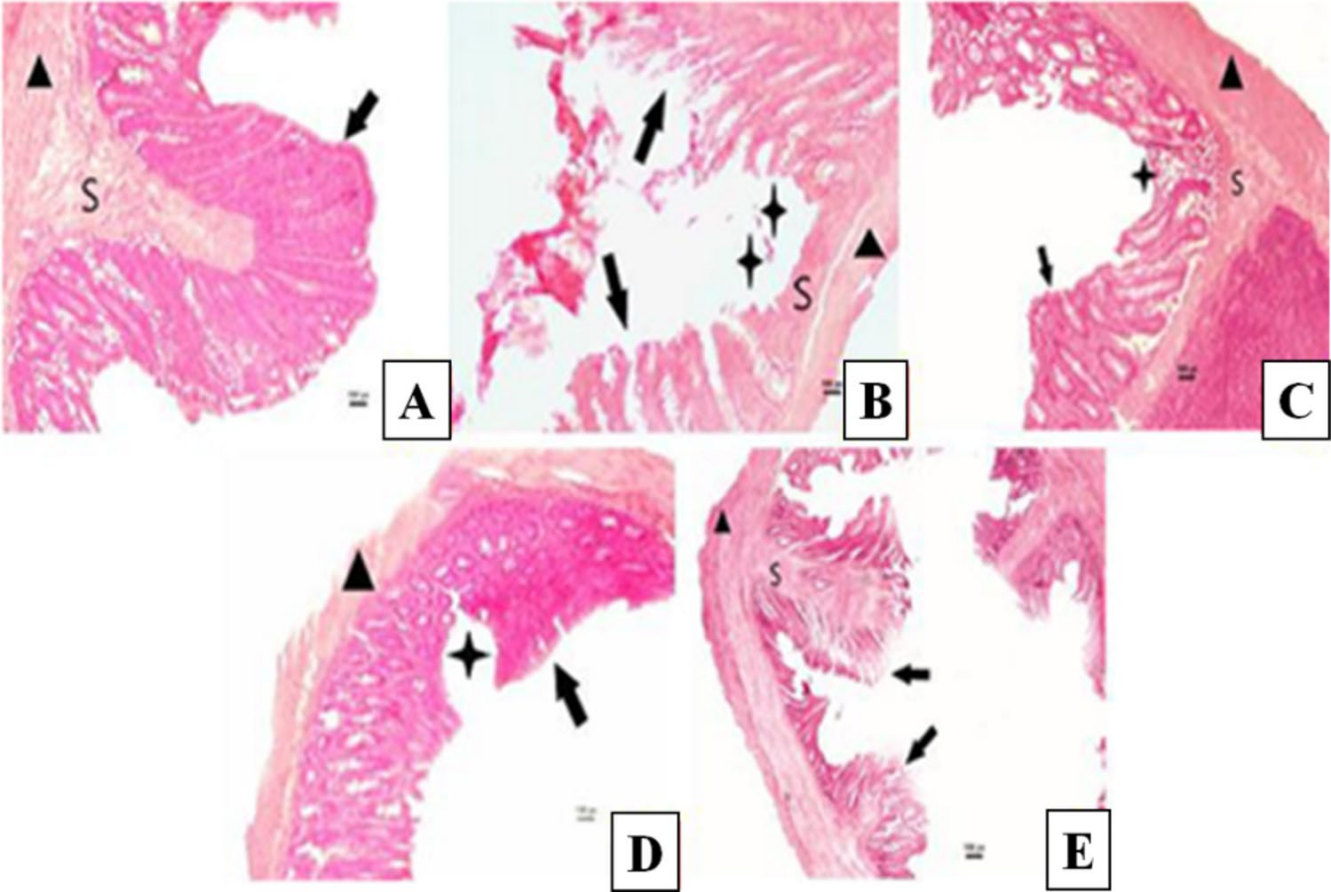
Histological evaluation of the small intestine tissue: intestinal permeability. Control group (**A**), stress group (**B**), bupropion + stress group (**C**), venlafaxine + stress group (**D**), LGG + stress group (**E**), (bar: 100.0μm, 20X, HE). **S**: Submucosa, ▲ : Muscularis layer, ⬆: Villus layer, ⋆ : Ulceration area

**Fig. 8:**
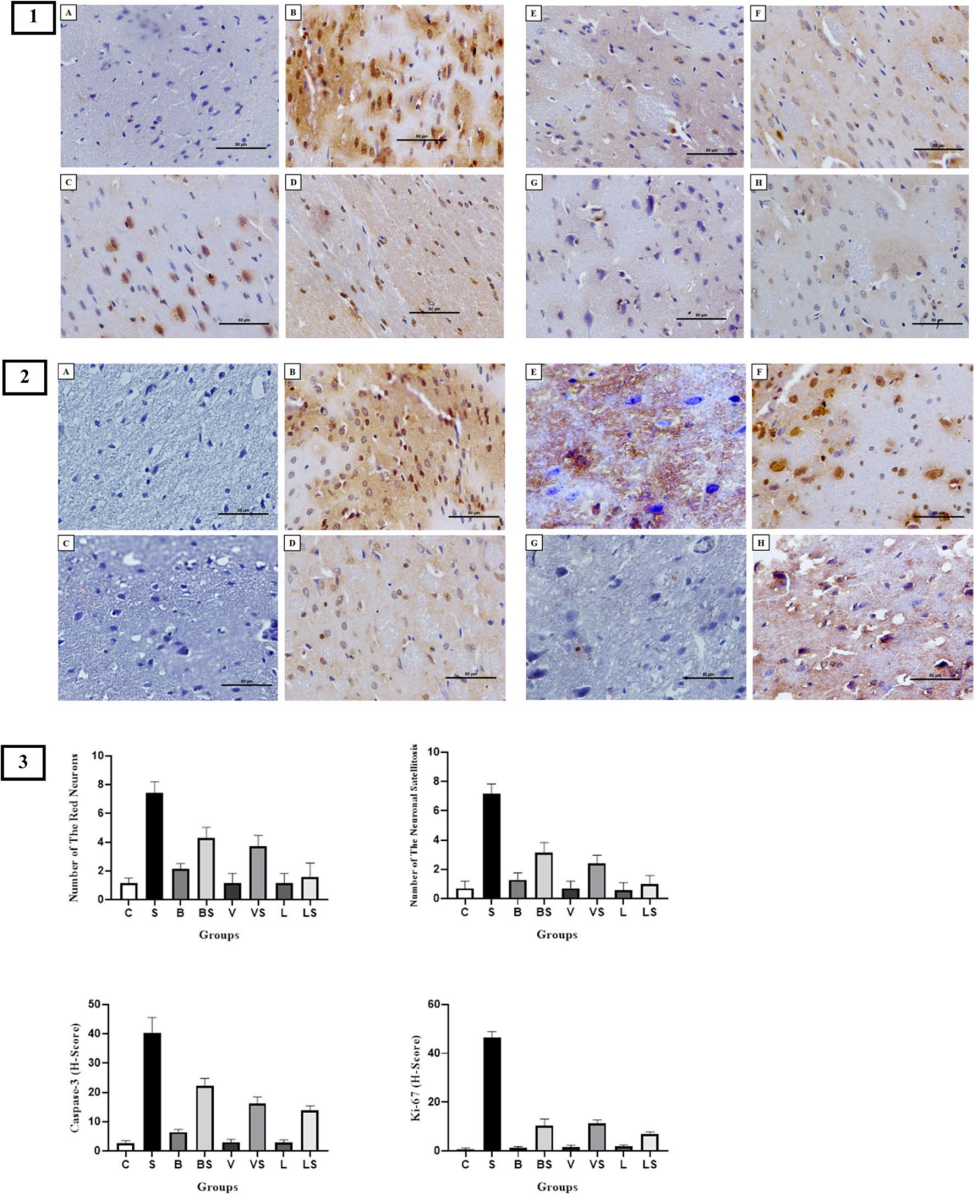
1. Caspase-3 activity in the cortex tissue, 2. Ki-67 activity in the cortex tissue, (bar: 50.0μm, 40X), 3. Graphical representation of histopathology results, control group (**A**), stress group (**B**), bupropion group (**C**), bupropion + stress group (**D**), venlafaxine group (**E**), venlafaxine + stress group (**F**), LGG group (**G**), LGG + stress group (**H**)

In the present study, preserved structures of villus, submucosa, and muscularis layers were observed in the C group. Ulceration areas were seen, and microvilli layers were not observed in high structure in the S group; this shows that intestinal permeability increased in the stress group. Ulceration areas extending down to the submucosa layer were observed in the BS group, and sparsed epithelial layers were also generally observed. Damaged epithelial areas were seen; although not down to the submucosal layer in the VS group, additionally, sparsed villus structures were seen. Although the histological structure was similar to the control group in the LS group, deconstructed and sparsed villus structures were seen compared to the C group (Fig. 7 and 8).

## Discussion

Depression is a mental illness that affects how a person thinks and feels, with symptoms such as prolonged sadness and feeling of helplessness. The intestinal barrier plays a crucial role in the progression of depression [99]. There are several experimental models of depression to evaluate depressive-like behaviors. The chronic unpredictable mild stress model is the most common and mostly preferred depression model [100]. In this study, the chronic unpredictable mild stress model was used; the therapeutic effects of probiotic *Lactobacillus rhamnosus* GG and antidepressant drugs bupropion and venlafaxine were investigated in stressed and non-stressed rats and the results were compared.

In stressed or depressed people, chronic stress causes increased appetite and increased body weight or decreased appetite and loss of body weight [101]. Many studies that used experimental depression models have shown that chronic stress causes a decrease in body weight [102, 103]. Studies conducted in rats have shown that bupropion has no effect on, and another study found that bupropion treatment reduced food intake [104–106]. It has been reported that venlafaxine treatment prevented weight loss caused by chronic unpredictable mild stress [107], and another study showed that venlafaxine prevented weight loss caused by depression [108]. It has been demonstrated that *Lactobacillus rhamnosus* GG prevents oxidative stress-induced weight loss [109]. *Lactobacillus rhamnosus* NCDC17 bacterium prevented body weight gain; in another study, it was shown that *Lactobacillus rhamnosus* MTCC:5957, *Lactobacillus rhamnosus* MTCC:5897 bacterium prevented streptozotocin-induced weight loss [110, 111]. Our results were similar to the literature, showing that LGG reduces weight loss during stress. Chronic stress causes irregularities in mood and energy homeostasis and increases vulnerability to anxiety, depression, and other mood disorders [112]. Depression is associated with weight loss and decreased appetite; MC4R signaling has an impact on feeding behavior, pain, drug addiction, regulation of HPA axis activity, emotional states, anxiety, and depression. MC4R signaling increases anxiety-like behavior [113, 114]. It has been observed that the MC4R expression level increased in transgenic C57BL/6 mice exposed to chronic restraint stress [115]. The increased MC4R expression level in the hippocampus in the S and BS groups compared to the C group may have caused the decrease in body weight. Because increasing MC4R expression level induces consume energy and leads to body weight loss. Other factors except MC4R activity may have affected body weight gain in the B and V groups. The lowest decrease in body weight was seen in the LS and V groups. In the LS group, LGG significantly reduced body weight loss by reducing the effect of chronic stress. While LGG, bupropion, and venlafaxine led to an increase in body weight in non-stressed rats, bupropion and venlafaxine had no effect on reversing the stress-induced decrease in body weight in stressed rats.

The sucrose preference test is one of the most common tests performed to evaluate anhedonia. Sucrose is used as a reward due to its effects on the dopamine system. Anhedonia, the most important symptom of depression, the loss of pleasure from rewarding or enjoyable activities, is a core symptom of depression in humans. Decreased sucrose consumption is a biomarker of anhedonia [116–120]. In the studies, it was observed that chronic stress decreased the percentage of sucrose preference in rats and the percentage of sucrose preference decreased to 60% in mice exposed to chronic stress [116, 121]. The results of studies performed in mice and rats showed that bupropion treatment increased stress-induced decreased sucrose consumption [122, 123]. In rats exposed to chronic mild stress, venlafaxine led to a significant increase in sucrose consumption compared to the untreated stress group [124]. *Lactobacillus reuteri* supplementation in C57BL/6 mice and *Lactobacillus helveticus* NS8 supplementation in rats increased sucrose consumption that stress-induced decreased [125, 126]. Norepinephrine, dopamine, and serotonin deficiency play a role in the emergence of anhedonia. Bupropion, venlafaxine, and LGG were effective in reversing the effects of chronic stress that causes anhedonia. In stressed rats, venlafaxine and LGG increased DRD1 expression; bupropion, venlafaxine, and LGG increased the ADRA-2A and 5-HT1A expression. These effects helped to reduce the level of anhedonia caused by chronic stress, and these results suggest that bupropion, venlafaxine, and the probiotic bacterium LGG may relieve symptoms of depression. The results we found coincide with the results of the previous studies.

The elevated plus maze test is the most preferred anxiety test to develop natural and unconditioned anxiety. Due to increasing anxiety, the time the experimental animals spend in the closed arm, the number of times entering the closed arm, and the percentage of time spent in the closed arm increase [84]. In an anxiety and depression study, the time spent by anxious rats in open arms was less than the time spent in closed arms [127], and in another study, the time spent by rats exposed to chronic stress in open arms was more than the time spent by rats in the control group in open arms [128]. Acute bupropion administration in mice increased the time spent in open arms [129], and in a study investigating the effects of bupropion on aggression and anxiety in OF1 male mice, no significant difference was found between bupropion-treated mice and control mice in terms of the percentage of entries into open arms or the time spent in open arms [130]. In mice under restraint stress, venlafaxine treatment increased the stress-induced decrease in time spent in open arms [131], and another study reported that venlafaxine treatment increased the time spent in open arms in rats exposed to chronic mild stress [132]. *Lactobacillus rhamnosus* JB-1 increased the time male BALB/c mice spent in open arms [133]; in another study, it was shown that *Lactobacillus brevis* ProGA28 bacterium increased the time Wistar-Kyoto mice spent in open arms [134]. LGG was more effective in the stressful condition than in the non-stressful condition according to our elevated plus maze test results. Our study results are in line with the literature.

The forced swim test is widely used in basic and preclinical research to evaluate the effectiveness of antidepressant medications and the effects of various behavioral and neurobiological manipulations. It enables despair behavior to emerge; the experimental animal loses hope of escape from a stressful environment [134, 136]. The forced swim test shows strong sensitivity to monoamine changes. FST represents a very specific set of stress-induced behaviors that have no direct, empirical relationship to symptoms of depression in humans and also this test is extremely sensitive to changes in monoamine levels. FST can also be used to investigate the genetic and neurobiological mechanisms underlying antidepressant and stress responses [137, 138]. A previous study reported that in rats exposed to chronic unpredictable stress, immobility time increased in the stressed rats compared to the control group [139]. In depressed male albino mice, bupropion reduced immobility time in the forced swim test [71]. Immobility time was reduced in venlafaxine-treated mice [140]. *Lacticaseibacillus rhamnosus* GG reduced immobility time in mouse with chronic unpredictable mild stress-induced depression [141]. Our results show that bupropion, venlafaxine, and LGG probiotic bacterium are effective in reversing depressive behaviors caused by chronic stress, and the results are correlated with the literature.

The social interaction test performed to examine social behavioral responses is preferred because it enables the evaluation of anxiety-like behaviors or depressed-like behaviors and locomotor activity. This test is a very useful model for the evaluation of anxiolytic compounds [85, 86, 90, 142]. Social interaction times in rats exposed to stress decreased compared to the control group [143]. In another study, it was shown that the interaction time with stranger partners decreased in rats exposed to chronic stress, and the time spent in the center, right, and left chambers was more than in the control group [144]. According to the results of the experimental depression study, bupropion did not affect the social interaction time in mice when there was only one stranger partner, but when the second stranger partner existed in the environment, it was shown that it increased the social interaction time compared to the control group and did not affect discovery behaviors [145]. Bupropion increased locomotor activity in mice but had no effect on locomotor activity at lower doses [146]. In an anxiety study, venlafaxine was found to have no effect on locomotor activity [147]. Another study showed that venlafaxine treatment reversed the stress-induced decrease in locomotor activity but had no effect on social interaction in Sprague–Dawley male rats [148]. It was observed that venlafaxine increased the number of rearing in rats compared to the control group [149]. *Lactobacillus helveticus* CCFM1076 significantly improved social interaction [150]. It has been shown that *Lactobacillus helveticus* R0052 and *Bifidobacterium longum* R0175 do not affect social interaction times in control groups but increase social interaction times in depressed rats and have no effect on rearing numbers [151]. LGG was effective in increasing interaction time. In stressed groups, only the probiotic bacterium LGG was able to reverse the decrease in social interaction time and locomotor activity caused by stress. The results we found are generally similar to the results of the studies in literature.

BDNF is the most widely distributed neurotrophin in the CNS; it is expressed at the highest levels in both the cerebral cortex and hippocampus. BDNF plays important roles in supporting the development, repair, neuronal growth and survival of CNS neurons, and plasticity of neurons, and is crucial for maintaining and regulating the structure and function of neurons throughout life. Decreased BNDF level in the hippocampus results in decreased proliferation of hippocampal neurons and decreased BDNF levels play a role in the pathogenesis of depression. BDNF was decreased in the serum and amygdala of patients with depression. Repeated stress exposure has been found to reduce hippocampal BDNF levels in rats. In adult rats, the hippocampus is the brain region having the highest levels of BDNF mRNA and protein, and a decrease in BDNF levels was observed in chronically stressed rats [152–155]. It was shown that chronic bupropion treatment significantly decreased BDNF expression in the rat hippocampus [156]. In one study, it was observed that bupropion treatment increased the BDNF level in mice exposed to tail suspension stress, in another study performed with the same stress model reported that bupropion treatment increased the BDNF level in the limbic system [157, 158]. Venlafaxine treatment increased BDNF expression in the dentate gyrus of rats exposed to chronic unpredictable stress [159]. In vitro study showed that *Lactobacillus rhamnosus* GG bacterium significantly increased neuronal BDNF mRNA expression compared to the control group 6 h after treatment, but there was a decrease in BDNF mRNA expression compared to the control group after 24 h [160]. In mice exposed to chronic unpredictable mild stress, *Lactobacillus rhamnosus* 4B15 significantly increased BDNF expression compared to the stress group [161]. Our results showed that chronic treatment with bupropion, venlafaxine, and LGG was effective in increasing the level of BDNF which decreased as a result of chronic stress.

Neurotransmitters and neurohormonal pathways play a major role in the pathophysiology of depression. Deficiency of serotonin (5-hydroxytryptamine; 5-HT), one of the monoamine neurotransmitters, is associated with anxiety, obsessions, and compulsions. 5-HT1A is the most abundant serotonin receptor in the hippocampus. Preclinical and clinical studies have proven that the 5-HT1A receptor, which is abundant in the limbic system, plays an important role in the pathophysiology of mood disorders. Chronic stress causes to decrease in the amount of 5-HT1A receptors. Scientific studies have revealed that the amount of 5-HT1A receptors decreases in the hippocampus in depressed and suicidal patients, and in experimental animals with chronic stress-induced depression. Antidepressant drug treatment has been found to upregulate the electrophysiological response of the hippocampal 5-HT1A receptor [162–165]. It was found that 5-HT1A expression in the CA1, CA3, and dentate gyrus regions of the hippocampus in rats exposed to chronic stress decreased compared to the control group [166]. Short- and long-term treatment with bupropion in mice doubled the mean firing rate of 5-HT neurons compared to the control group [167]. In another study, unlike the selective serotonin reuptake inhibitor paroxetine, bupropion increased tonic activation of 5-HT1A receptors in the hippocampus [168]. Venlafaxine treatment significantly increased the level of 5-HT in the brain in stressed rats [169]. In a study performed in mice, *Lactobacillus paracasei* PS23 supplementation significantly increased 5-HT levels in the hippocampus [170], and in another study, *Lactobacillus plantarum* PS128 increased 5-HT levels in the prefrontal cortex and hippocampus in 8-week-old mice exposed to early life stress [51]. According to our results, bupropion and LGG were found effective in increasing 5-HT1A receptor expression levels in stressful situations.

Another topic we discussed in our study, the mesolimbic dopaminergic pathway extends from the ventral tegmental area to the prefrontal cortex and limbic region, and dopamine regulates motivational and emotional behavior through this pathway. Disruption of this pathway can cause anhedonia which can lead to decreased motivation and depression [171, 172]. Dopamine also affects intestinal motility and intestinal secretion, and the dopamine system is involved in the function of the microbiota-gut-brain axis [173]. Dopamine causes inhibitory effects on the progression of neuroinflammation by inhibiting NLRP3 inflammasome activation through activation of the DRD1 receptor [174, 175]. DRD1 is the most abundant dopamine receptor in the CNS and is a functionally specific marker of the dopamine system. Decreased DRD1 expression levels are found in patients with emotional disorders including depression. In C57BL/6 mice exposed to chronic immobilization stress, a decrease in DRD1 expression levels was observed in the nucleus accumbens and prefrontal cortex compared to the control group [176, 177]. Stress-induced DRD1 downregulation was observed in the hippocampus of mice exposed to chronic mild stress [178]. Dose-dependent bupropion treatment (10, 20, and 40 mg/kg i.p.) increased brain dopamine levels in mice [71], and another study showed that DRD1 expression in mice treated with bupropion was at a level similar to the control group [179]. Fluoxetine treatment in mice caused an increase in DRD1 expression level in the dentate gyrus [180], and venlafaxine treatment in rats caused an increase in dopamine levels in the frontal cortex [181]. As a result of LGG treatment in adult rats, an increase in dopamine levels was observed in the brainstem and lower brain region (subconscious region/subcortex) in the bacterial group compared to the control group [64]. In the current study, venlafaxine and LGG were found effective in increasing DRD1 expression levels under stressful conditions.

Norepinephrine plays a role in functions such as stress reactivity, resilience, and arousal and reduces synaptic inhibition in the hippocampus. Norepinephrine signaling is thought to be dysregulated in many psychiatric disorders [182, 183]. Most findings regarding the norepinephrine system and the role of antidepressants have focused on α2 receptors. α2 adrenoreceptors are located in norepinephrine neuronal cell bodies, both presynaptic and postsynaptic neurons, where they mediate an inhibitory role in the central and peripheral nervous systems [184, 185]. α2-adrenergic receptors, especially subtype A (ADRA-2A), are widely distributed in the brain, are primarily responsible for central noradrenergic functions, and play a critical role as an autoreceptor in the regulation of neuronal firing and the release of norepinephrine and other neurotransmitters. α2A-AR expression is required for normal behavior [186–188]. In a study on neurotransmitter system genes in the ventral tegmental region of depressed male mice, the gene related to ADRA-2A was found to be downregulated in depressed mice [189]. It has been observed that ADRα2 expression is significantly reduced in chronically stressed rats and also norepinephrine levels in the hippocampus are reduced compared to the control group [190]. It was found that 2 weeks of bupropion treatment in rats increased synaptic levels of norepinephrine in the hippocampus [191]. It was shown that fluoxetine treatment in stressed rats increased the stress-induced decreased norepinephrine levels in the hippocampus to a level similar to the control group and neither acute nor chronic fluoxetine treatment changed the basal level of norepinephrine in the hippocampus [192, 193]. *Lactobacillus plantarum* PS128 supplementation in naive mice does not cause an increase in striatal norepinephrine levels compared to the control group [194]. An increase in ADRA-2A expression level which decreased due to chronic stress, was observed in all stress groups administered treatment. Venlafaxine and LGG were more effective than bupropion in increasing ADRA-2A expression levels under stress conditions.

GABA, the main inhibitory neurotransmitter in the brain, is responsible for both fine-tuning and overall control of excitatory transmission, stress, and depression impair the function of GABA [195]. It is becoming increasingly clear that disruption of the GABAergic system and thus the excitation-inhibition balance may contribute to the pathophysiology of many psychiatric disorders including depression [196]. GABA released from GABAergic interneurons shows its effect mainly through GABA-A receptors [197]. GABA-A receptors are sensitive to stress-related changes in the environment in early life and adulthood; these receptors are involved in the regulation or modulation of emotional behavior. The effect of GABA on emotional behavior occurs through the activation of specific GABA-A receptor subunits [198, 199]. While the GABA-A α1 receptor expression level in the hippocampus of rats exposed to juvenile stressors was not affected in the short term, its expression level was decreased compared to the control group in the long term [200]. Other studies have shown that the GABA-A α1 receptor expression level decreased in the hippocampus of mice exposed to repeated swim stress [201] and in the prefrontal cortex and hippocampus of rats exposed to restraint stress [202]. Venlafaxine treatment increased GABA levels in the hippocampus of rats exposed to early-life stress [203], and LGG treatment increased GABA-A α1/α2 receptor expression levels in the hippocampus of male rats exposed to early-life stress [204]. Our results show that *Lactobacillus rhamnosus* GG causes an antidepressive effect; however, bupropion and venlafaxine are not effective enough to increase the expression level compared to the LGG in stressful situation.

The role of the endocannabinoid system and its receptors (CB1 and CB2) in the neurobiology and management of mood disorders has drawn considerable interest [205]. The CB1 receptor is expressed on central and peripheral nerve terminals; it is involved in fine-tuning excitatory and inhibitory neurotransmission [206, 207]. The fact that CB1 agonists cause anxiolytic and antidepressant-like effects makes the endocannabinoid system a promising target for antidepressant drugs [31]. Activation of the CB1 receptors prevents the release of neurotransmitters that play a role in anxiety and depression [208]. Chronic stress leads to a decrease in both the CB1 receptor density and endocannabinoid content in the hippocampus, whereas chronic treatment with a tricyclic antidepressant increases endocannabinoid activity in the hippocampus. The increase in activation of the CB1 receptors in the dentate gyrus of the hippocampus causes an antidepressant-like effect [209]. In a study, CNR1 expression level was observed to decrease in the hippocampus of depressed rats [210], and another study showed that cannabinoid receptor activation prevented the effects of chronic mild stress in rats [211]. Bupropion reduced the protein levels of the CB1 in the prefrontal cortex of ethanol-exposed rats [212]. CNR1 expression level increased in depressed patients administered citalopram, duloxetine, fluoxetine, mirtazapine, or venlafaxine treatment [213]. Probiotic bacterial mix increased CNR1 expression level in zebrafish [214]. In our study, bupropion, venlafaxine, and LGG treatment were effective in reversing the chronic stress-induced decrease in CNR1 expression levels, and the treatment provided an antidepressant effect. *Lactobacillus rhamnosus* GG has the strongest effect in increasing the CNR1 expression level.

Exposure to stress causes the release of corticosteroids through the HPA axis, which represents the major neuroendocrine stress response system. Prolonged or excessive HPA axis activity following chronic stress can overwhelm the individual’s allostatic load and subsequently lead to the development of psychiatric and somatic disorders. The effect of cortisol, including feedback regulation of the HPA axis, is mediated by both GR and MR which is expressed at high density in limbic brain regions that play a role in the processing of emotional information. Hippocampal MRs (NR3C2 receptors) are important for the control of inhibitory tone on the HPA axis. Dysfunction of the HPA axis may play a key role in triggering depression [9, 33, 215, 216]. In an early-life experimental stress model, a decrease in the expression level of the NR3C2 receptor was observed in the hippocampus of rats exposed to stress [217]. It has been shown that GR expressions increase but MR expressions decrease in the hippocampus of rats exposed to chronic stress [218]. Chronic treatment with bupropion in rats reduced immobility time in the forced swim test and therefore suppressed HPA activity [219]. Venlafaxine treatment reversed the deleterious effects of chronic stress by modulating HPA activity [203]. In zebrafish, LGG treatment increased MR expression in the hippocampus [220]. It was observed that probiotic LGG treatment in male rats exposed to early-life stress could not prevent the stress-induced decrease in MR expression in the hippocampus [204]. In our study bupropion, venlafaxine, and LGG were not effective in increasing decreased MR expression but LGG reduced the downregulation level.

The NLRP3 inflammasome is a cytosolic pattern recognition receptor and a molecular mechanism that converts psychologically stressful stimuli into inflammatory responses; it plays a role in inducing cell death processes such as pyroptosis, apoptosis, and necroptosis [221–223]. New strategies in terms of the development of effective treatments for depression in humans may be to restore microglial homeostasis in the hippocampus and reduce or inhibit NLRP3 inflammasome activation [41]. Dysfunction in the autophagy mechanism, a homeostatic cellular process that can sense intracellular stress and respond rapidly to cope with damage, is associated with various neurodegenerative and mood disorders, especially depression. Autophagy plays an active role in the elimination of proinflammatory cytokines; it thus serves as a central fulcrum that balances inflammatory responses. In the development and treatment of depression, autophagy may interact with the inflammatory process. While previous scientific studies have shown that autophagy inhibits the NLRP3 inflammasome, recent studies have shown that autophagy may play a role in NLRP3 inflammasome activity in some cases, and the NLRP3 inflammasome can also affect autophagy [224–227]. It has been observed that interleukin-1β production and NLRP3 inflammasome activation increased in the brain of depressed rats exposed to chronic mild stress [228], and another study showed that chronic stress activated the NLRP3 inflammasome in the rat hippocampus [229]. Bupropion treatment reduced the production of TNF-α, IL-1β, and IFN-γ in mice [230]. Venlafaxine treatment normalized changes in NLRP3 levels in the hippocampus of rat pups exposed to prenatal stress [231]. *Lactobacillus rhamnosus* GR-1 ameliorated inflammation and cell damage by attenuating NLRP3 inflammasome activation [232]. LGG activated the NLRP3 inflammasome and antiviral responses in human macrophages [233]. In the present study, bupropion provided the most effective treatment in reducing the effects of chronic stress by highly suppressing NLRP3 expression in stressed rats. Venlafaxine treatment was effective in reducing NLRP3 expression in stressed rats compared to untreated stressed rats. LGG treatment was ineffective in reducing stress-induced increased NLRP3 expression. LGG probably increased the NLRP3 expression level to activate the autophagy mechanism and provided a protective effect against damage caused by chronic stress.

The NOD1 receptor is an intracellular pattern recognition receptor that mediates the recognition of specific parts of the peptidoglycan layer by the host and plays an important role in maintaining intestinal homeostasis by enabling the formation of protective immune responses against bacteria. Intestinal homeostasis is achieved by improving epithelial barrier function and supporting resistance to pathogens. The NOD1 receptor supports intestinal homeostasis by modulating antimicrobial peptides, proinflammatory cytokines, autophagy, and adaptive immunity [35, 234–236]. The microbiota plays a role in modulating the gastrointestinal tract and bacterial infection; it is also an important contributor to neurodevelopment. Dysbiosis triggers psychiatric disorders and behavioral deviations. The intestinal epithelial NOD1 receptor, a potential regulator of the connections between the microbiota and the nervous system, regulates sensitivity to cognitive impairment and anxiety-like and depressive-like behaviors [35, 234]. In a study performed in mice, it was observed that NOD1 receptor expression increased in the stress group compared to the control group [237]. *Lactobacillus rhamnosus* ATCC 7469 probiotic bacterium increased NOD1 mRNA expression in both healthy cells and infected cells [238], and in another study, *Lactobacillus rhamnosus* ATCC 7469 increased NOD1 mRNA expression in the jejunum and ileum in infected experimental animals [239]. In our study, in the treated stressed groups, bupropion and LGG were effective in increasing the chronic stress-induced decreased NOD1 receptor expression level in intestinal tissue, but venlafaxine was not effective in reversing the effect of chronic stress.

Apoptosis, which is programmed cell death, is an important process that has an important role in neuronal and glial death and neurodegeneration [240, 241]. Clinical and preclinical studies have shown that apoptotic pathways involved in the mechanisms of stress-induced psychopathology cause adverse effects on neuronal survival and plasticity in stress-induced depression [242]. Chronic stress stimulates apoptosis in the cerebral cortex and increases the expression levels of caspase proteins [37]. Chronic stress causes atrophy in dendrites and dendritic spines and a decrease in neurogenesis. Increasing evidence shows that depression is a progressive disorder of neurodegeneration and neuronal damage is the main pathology of depression. It has been shown that chronic unpredictable mild stress leads to increased neuronal apoptosis and caspase-3 expression level in rats [243]. Bupropion treatment decreased neurodegeneration level in the cerebral cortex [244]. Venlafaxine treatment decreased neuronal apoptosis level in depressed rats [245], and another study showed that venlafaxine treatment in depressed rats decreased the activity of caspase-3 that is an apoptotic and inflammatory biomarker [246]. *Lactobacillus rhamnosus* 4B15 decreased caspase-3 expression level in mice exposed to chronic stress [161]. LGG was approximately 2 times more effective than the antidepressant drugs bupropion and venlafaxine in protecting the brain against chronic stress-induced neuronal damage.

Depression is usually caused by stress and is considered a neuropsychiatric disease associated with neuronal damage in specific brain regions [247]. Neuroinflammation is associated with a number of neurological diseases and is an important factor in the formation of neuronal damage [248]. Glial functions play a role in the neuropathology of psychiatric disorders such as depression and manic-depressive (bipolar) illness [249]. Neuroinflammation and glial activation are major factors involved in the etiology of most neurodegenerative diseases. Upregulation of glial activities is an indicator of stress and often indicates degeneration and disruption of chemical balance. Assessment of glial activation is determined by Ki-67 activity [42]. Neurotransmitter and receptor systems that mediate synaptic plasticity also play a role in the activation of glial cells [250]. Aggregations of glial cells called neuronal satellitosis have been identified in healthy peripheral nerve tissue surrounding both the cell body and dendrites of neurons. Glial cells can aggregate in physiological states, but this is more commonly a histological marker of various pathological conditions in the CNS [251]. Microglial activation has been associated with various diseases in the clinic. In particular, microglial activity has been seen in various brain regions where significant neurodegeneration occurs in various neurodegenerative disorders. It has been shown that emotional or physical stress causes neuroinflammation by leading to microglial activation in rats [252]. Bupropion did not inhibit or reduce the function of activated microglia in murine [253]. A study in C57BL/6 mice showed that venlafaxine treatment inhibited microglia and astrocyte activation [254]. *Lactobacillus rhamnosus* PTCC1637 reduced lipopolysaccharide-induced neuroinflammation in rats [255]. The probiotic mixture consisting of *Bifidobacterium animalis ssp. lactis* Bb12 and *Lactobacillus rhamnosus* GG caused an anti-inflammatory effect by reducing microglial activation [256]. Bupropion, venlafaxine, and LGG were highly effective in reducing glial activation and subsequently preventing neuronal damage by reducing neuroinflammation, LGG probiotic bacterium showed the strongest effect.

The intestinal barrier, a complex structure consisting of multiple layers of defense barriers, regulates transport and host defense mechanisms at the mucosal interface with the external environment to maintain intestinal homeostasis [99, 257]. Chronic psychological stress causes various changes in intestinal homeostasis that can impair health. Chronic stress has significant effects on intestinal physiology and pathophysiology, including changes in gastrointestinal motility, increased perception of visceral pain (hyperalgesia), and impaired intestinal barrier function. Chronic psychological stress activates the HPA axis which affects various physiological functions of the gastrointestinal tract including intestinal permeability and barrier function, and the activated HPA axis increases epithelial permeability [257–259]. Changes in intestinal permeability lead to the formation of proinflammatory cytokines. The intestinal barrier plays a crucial role in the progression of depression. Intestinal epithelial barrier dysfunction (leaky gut) and increased intestinal permeability leading to autoimmunity (during depression) have been observed in patients with depression. Gut symptomatology and mental health are closely interrelated. Approximately 60% of patients with anxiety and depression are diagnosed with intestinal dysfunction [43, 99, 260, 261]. Impaired intestinal permeability was observed in mice after 6 weeks of unpredictable chronic mild stress exposure [262]. Bupropion treatment reduced intestinal damage through inhibition of TNF-α in rats [263]. Venlafaxine treatment decreased intestinal permeability by increasing zonula occludens-1 and occludin expressions in stressed male rats [264]. LGG increases ZO-1, occludin, and Bcl-2 mRNA levels and decreases Bax mRNA levels in jejunal mucosal tissue; this indicates that LGG supplementation may decrease intestinal permeability by reducing apoptosis [265]. LGG reduced intestinal permeability in male C57BL/6N mice and rats [266, 267]. According to our results, it was seen that bupropion and venlafaxine partially decreased intestinal permeability caused by chronic stress, and LGG had a stronger effect on protecting the intestinal barrier from chronic stress-induced damage.

According to the results we found in the study, briefly, LGG treatment increased the BDNF level, 5-HT1A, DRD1, ADRA-2A, GABA-A α1, CNR1, and NOD1 receptor expression levels in the hippocampus and also reduced the neurodegeneration level and glial cell activity in the cerebral cortex in chronically stressed rats in the LS group. The increase in BDNF level increases the survival of neurons and thus may have led to an increase in the number of receptors having increased expression and increased GABA release. Mutual interaction between monoaminergic neurons may have increased 5-HT1A, DRD1, and ADRA-2A receptor expression levels. Increased receptor expression levels of neurotransmitters such as serotonin, dopamine, norepinephrine, and GABA, which have important roles in the pathology of depression, are important in alleviating depression symptoms. The decrease in glial cell activation reduced chronic stress-induced neuronal death, leading to a limitation of depression formation. Increased CNR1 expression level may have caused an increase in monoamine and BDNF levels, also a sedative and analgesic effect. Activation of the endocannabinoid system led to antidepressant effects, and LGG-induced CNR1 receptor activation was demonstrated for the first time in our study. The increased NOD1 receptor expression level due to the effect of increasing serotonin levels may have caused a decrease in the release of proinflammatory cytokines and activation of the autophagy mechanism. Additionally, the increase in NLRP3 receptor expression level may have caused the autophagy mechanism to be activated. The decrease in intestinal permeability and the combined result of all these effects provided by LGG treatment may have led to a decrease in the level of neurodegeneration and glial cell activity and a decrease in depressive-like behaviors, as seen in the results of behavioral tests. Bupropion treatment in chronically stressed rats in the BS group may have caused a decrease in the level of neurodegeneration and glial cell activity by increasing the BDNF level, ADRA-2A, CNR1, and NOD1 receptor expression levels and decreasing the NLRP3 receptor expression level and, consequently, a decrease in intestinal permeability and depressive-like behaviors. Venlafaxine treatment in chronically stressed rats in the VS group may have caused a decrease in the level of neurodegeneration and glial cell activity by increasing the BDNF level, DRD1, ADRA-2A, GABA-A α1, and CNR1 expression levels and decreasing the MC4R and NLRP3 receptor expression level and, consequently, decreased intestinal permeability and depressive-like behaviors. Most likely, bupropion and venlafaxine showed their effects in the gastrointestinal tract through the efferent fibers of the vagus nerve, and *Lactobacillus rhamnosus* GG showed their effects in the brain through the afferent fibers of the vagus nerve, humoral, immune, and metabolic pathways. In line with our results, it has been demonstrated that bupropion, venlafaxine, and *Lactobacillus rhamnosus* GG (ATCC 53103) probiotic bacterium cause antidepressant effects through various mechanisms.

## Conclusion

In conclusion, it was revealed for the first time in this study that the probiotic bacterium *Lactobacillus rhamnosus* GG (ATCC 53103) has antidepressant properties and was found more effective than the antidepressant drugs bupropion and venlafaxine. LGG is a potential psychobiotic bacterium and can be useful in depression treatment. LGG consumption can provide benefits for mental health. Clinical trials are needed to see the antidepressant effects of *Lactobacillus rhamnosus* GG (ATCC 53103) in humans.

## Data Availability

No datasets were generated or analysed during the current study.

## References

[1] Pozos-Radillo BE, de Lourdes Preciado-Serrano M, Acosta-Fernández M, de los Ángeles Aguilera-Velasco M, Delgado-García DD. Academic stress as a predictor of chronic stress in university students. Psicología educativa. 2014 Jun 1;20(1):47-52.

[2] Lenow JK, Constantino SM, Daw ND, Phelps EA (2017) Chronic and acute stress promote overexploitation in serial decision making. J Neurosci 37(23):5681–5689 28483979 10.1523/JNEUROSCI.3618-16.2017 PMC5469305

[3] Selye H (1956) What is stress. Metabolism 5(5):525–30 13358567

[4] Yağar SD Stres Ve Sağlık Çalışanları 2009–2018 Yılları Arasında Türkiye’de Yapılmış Çalışmaların İncelenmesi. Ankara Üniversitesi Sağlık Bilimleri Fakültesi (2022).

[5] D’Ambrosio F, Caggiano M, Schiavo L, Savarese G, Carpinelli L, Amato A, Iandolo A (2022) Chronic stress and depression in periodontitis and peri-implantitis: a narrative review on neurobiological, neurobehavioral and immune–microbiome interplays and clinical management implications. Dentistry Journal 10(3):49 35323251 10.3390/dj10030049 PMC8947556

[6] Liu HM, Li C, Cao B, Jiang Y, Han L, Xu R, ... Zhang D The molecular mechanism of chronic stress affecting the occurrence and development of breast cancer and potential drug therapy. *Translational oncology*, *15*(1), 101281 (2022). 10.1016/j.tranon.2021.101281 PMC8652015 34875482

[7] Gianaros PJ, Jennings JR, Sheu LK, Greer PJ, Kuller LH, Matthews KA (2007) Prospective reports of chronic life stress predict decreased grey matter volume in the hippocampus. Neuroimage 35(2):795–803 17275340 10.1016/j.neuroimage.2006.10.045 PMC1868546

[8] Hussenoeder FS, Conrad I, Pabst A, Luppa M, Stein J, Engel C, ... Riedel-Heller SG Different areas of chronic stress and their associations with depression. International journal of environmental research and public health, 19(14), 8773 (2022). 10.3390/ijerph19148773 PMC9315834 35886625

[9] Zhang S, Xu Z, Gao Y, Wu Y, Li Z, Liu H, Zhang C (2012) Bidirectional crosstalk between stress-induced gastric ulcer and depression under chronic stress. PLoS ONE 7(12):e51148 23251441 10.1371/journal.pone.0051148 PMC3521024

[10] Goodwin GM (2006) Depression and associated physical diseases and symptoms. Dialog Clinic Neurosci 8(2):259–65 10.31887/DCNS.2006.8.2/mgoodwin PMC3181771 16889110

[11] Pinto B, Conde T, Domingues I, Domingues MR (2022) Adaptation of lipid profiling in depression disease and treatment: a critical review. Int J Mol Sci 23(4):2032 35216147 10.3390/ijms23042032 PMC8874755

[12] Chen Y, Shen X, Feng J, Lei Z, Zhang W, Song X, Lv C (2022) Prevalence and predictors of depression among emergency physicians: a national cross-sectional study. BMC Psychiatry 22(1):1–8 35090424 10.1186/s12888-022-03687-8 PMC8795725

[13] Reus GZ, Dos Santos MAB, Strassi AP, Abelaira HM, Ceretta LB, Quevedo J (2017) Pathophysiological mechanisms involved in the relationship between diabetes and major depressive disorder. Life Sci 183:78–82 28676432 10.1016/j.lfs.2017.06.025

[14] Fasick V, Spengler RN, Samankan S, Nader ND, Ignatowski TA (2015) The hippocampus and TNF: common links between chronic pain and depression. Neurosci Biobehav Rev 53:139–159 25857253 10.1016/j.neubiorev.2015.03.014

[15] Dionisie V, Ciobanu AM, Toma VA, Manea MC, Baldea I, Olteanu D, Sevastre-Berghian A, Clichici S, Manea M, Riga S, Filip GA (2021) Escitalopram targets oxidative stress, caspase-3, BDNF and MeCP2 in the hippocampus and frontal cortex of a rat model of depression induced by chronic unpredictable mild stress. Int J Molecul Sci 22(14):7483 10.3390/ijms22147483 PMC8304451 34299103

[16] Fekadu N, Shibeshi W, Engidawork E (2017) Major depressive disorder: pathophysiology and clinical management. J Depress Anxiety 6(1):255–257

[17] Depression WH (2017) Other common mental disorders: global health estimates. Geneva: World Health Organization. Feb;24(1).

[18] Kiyohara C, Yoshimasu K (2009) Molecular epidemiology of major depressive disorder. Environ Health Prev Med 14:71–87 19568851 10.1007/s12199-008-0073-6 PMC2684780

[19] Depression WH (2017) Other common mental disorders: global health estimates. Geneva: World Health Organ 24(1).

[20] d de Anta L, Alvarez-Mon MA, Ortega MA, Salazar C, Donat-Vargas C, Santoma-Vilaclara J, Martin-Martinez M, Lahera G, Gutierrez-Rojas L, Rodriguez-Jimenez R, Quintero J. Areas of interest and social consideration of antidepressants on english tweets: a natural language processing classification study. J Personal Med 2022 Jan 25;12(2):155. 10.3390/jpm12020155 PMC8879287 35207644

[21] Landmark CJ, Henning O, Johannessen SI (2016) Proconvulsant effects of antidepressants—what is the current evidence? Epilepsy Behav 61:287–291 26926001 10.1016/j.yebeh.2016.01.029

[22] Holliday SM, Benfield P (1995) Venlafaxine: a review of its pharmacology and therapeutic potential in depression. Drugs 49:280–294 7729333 10.2165/00003495-199549020-00010

[23] Kim EJ, Felsovalyi K, Young LM, Shmelkov SV, Grunebaum MF, Cardozo T (2018) Molecular basis of atypicality of bupropion inferred from its receptor engagement in nervous system tissues. Psychopharmacology 235:2643–2650 29961917 10.1007/s00213-018-4958-9 PMC6132670

[24] Foley KF, DeSanty KP, Kast RE (2006) Bupropion: pharmacology and therapeutic applications. Expert Rev Neurother 6(9):1249–1265 17009913 10.1586/14737175.6.9.1249

[25] Cassano P, Fava M (2004) Tolerability issues during long-term treatment with antidepressants. Ann Clin Psychiatry 16(1):15–25 15147109 10.1080/10401230490281618

[26] Feighner JP (1999) Mechanism of action of antidepressant medications. J Clin Psychiatry 60(4):4–13 10086478

[27] Dagytė G (2010) The stressed brain: ınquiry into neurobiological changes associated with stress, depression and novel antidepressant treatment

[28] Anand KS, Dhikav V (2012) Hippocampus in health and disease: an overview. Ann Indian Acad Neurol 15(4):239 23349586 10.4103/0972-2327.104323 PMC3548359

[29] Alcocer-Gómez E, Casas-Barquero N, Williams MR, Romero-Guillena SL, Cañadas-Lozano D, Bullón P, Sánchez-Alcazar JA, Navarro-Pando JM, Cordero MD (2017) Antidepressants induce autophagy dependent-NLRP3-inflammasome inhibition in Major depressive disorder. Pharmacol Res 1(121):114–21 10.1016/j.phrs.2017.04.028 28465217

[30] Lommatzsch M, Zingler D, Schuhbaeck K, Schloetcke K, Zingler C, Schuff-Werner P, Virchow JC (2005) The impact of age, weight and gender on BDNF levels in human platelets and plasma. Neurobiol Aging 26(1):115–123 15585351 10.1016/j.neurobiolaging.2004.03.002

[31] Zhong P, Wang W, Pan B, Liu X, Zhang Z, Long JZ, Zhang HT, Cravatt BF, Liu QS (2014) Monoacylglycerol lipase inhibition blocks chronic stress-induced depressive-like behaviors via activation of mTOR signaling. Neuropsychopharmacology 39(7):1763–76 24476943 10.1038/npp.2014.24 PMC4023150

[32] Chaki S, Okuyama S (2005) Involvement of melanocortin-4 receptor in anxiety and depression. Peptides 26(10):1952–1964 15979204 10.1016/j.peptides.2004.11.029

[33] Wingenfeld K, Otte C (2019) Mineralocorticoid receptor function and cognition in health and disease. Psychoneuroendocrinology 105:25–35 30243757 10.1016/j.psyneuen.2018.09.010

[34] ter Heegde F, De Rijk RH, Vinkers CH (2015) The brain mineralocorticoid receptor and stress resilience. Psychoneuroendocrinology 52:92–110 25459896 10.1016/j.psyneuen.2014.10.022

[35] Pusceddu MM, Barboza M, Keogh CE, Schneider M, Stokes P, Sladek JA, Kim HJ, Torres-Fuentes C, Goldfild LR, Gillis SE, Brust-Mascher I (2019) Nod-like receptors are critical for gut–brain axis signalling in mice. J Physiol 597(24):5777–97 31652348 10.1113/JP278640 PMC6911019

[36] Fuchs E, Flügge G (1998) Stress, glucocorticoids and structural plasticity of the hippocampus. Neurosci Biobehav Rev 23(2):295–300 9884123 10.1016/s0149-7634(98)00031-1

[37] Bachis A, Cruz MI, Nosheny RL, Mocchetti I (2008) Chronic unpredictable stress promotes neuronal apoptosis in the cerebral cortex. Neurosci Lett 442(2):104–108 18621098 10.1016/j.neulet.2008.06.081 PMC2543936

[38] Zhao T, Wu D, Du J, Liu G, Ji G, Wang Z, Peng F, Man L, Zhou W, Hao A (2022) Folic acid attenuates glial activation in neonatal mice and improves adult mood disorders through epigenetic regulation. Front Pharmacol 7(13):818423 10.3389/fphar.2022.818423 PMC8859176 35197855

[39] Mechawar N, Savitz J (2016) Neuropathology of mood disorders: do we see the stigmata of inflammation? Transl Psychiatry 6(11):e946–e946 27824355 10.1038/tp.2016.212 PMC5314124

[40] Jia X, Gao Z, Hu H (2021) Microglia in depression: current perspectives. Science China Life Sciences 64:911–925 33068286 10.1007/s11427-020-1815-6

[41] Feng X, Zhao Y, Yang T, Song M, Wang C, Yao Y, Fan H (2019) Glucocorticoid-driven NLRP3 inflammasome activation in hippocampal microglia mediates chronic stress-induced depressive-like behaviors. Front Mol Neurosci 12:210 31555091 10.3389/fnmol.2019.00210 PMC6727781

[42] Ogundele OM, Omoaghe AO, Ajonijebu DC, Ojo AA, Fabiyi TD, Olajide OJ, Falode DT, Adeniyi PA (2014) Glia activation and its role in oxidative stress. Metabolic Brain Dis 29:483–93 10.1007/s11011-013-9446-7 24218104

[43] Liu L, Zhu G (2018) Gut–brain axis and mood disorder. Front Psych 9:223 10.3389/fpsyt.2018.00223 PMC5987167 29896129

[44] Mass M, Kubera M, Leunis JC (2008) The gut-brain barrier in major depression: intestinal mucosal dysfunction with an increased translocation of LPS from gram negative enterobacteria (leaky gut) plays a role in the inflammatory pathophysiology of depression. Neuroendocrinol Lett 29(1):117–124 18283240

[45] Skowron K, Budzyńska A, Wiktorczyk-Kapischke N, Chomacka K, Grudlewska-Buda K, Wilk M, Wałecka-Zacharska E, Andrzejewska M, Gospodarek-Komkowska E (2022) The role of psychobiotics in supporting the treatment of disturbances in the functioning of the nervous system—a systematic review. Int J Molecul Sci 23(14):7820 10.3390/ijms23147820 PMC9319704 35887166

[46] Yangılar F (2015) Probiyotik mikroorganizmaların biyokoruyucu özelliği. Uludağ Üniversitesi Mühendislik Fakültesi Dergisi 20(1):119–130

[47] Usta M, Urgancı N (2014) Çocukluk çağında probiyotik kullanımı. Güncel Pediatri 12(2):88–94

[48] Özbek B (2010) Probiyotikler: Biyolojik Terapi. Türk Mikrobiyoloji Cemiyeti Dergisi 40(4):207–218

[49] Velmani G (2010) Probiotics as potential therapies in human gastrointestinal health

[50] Sağdıç O, Küçüköner E, Özçelik S. Probiyotik ve prebiyotiklerin fonksiyonel özellikleri. Research in Agricultural Sciences. 2004;35(3-4).

[51] Liu YW, Liu WH, Wu CC, Juan YC, Wu YC, Tsai HP, Wang S, Tsai YC (2016) Psychotropic effects of Lactobacillus plantarum PS128 in early life-stressed and naïve adult mice. Brain Res 15(1631):1–2 10.1016/j.brainres.2015.11.018 26620542

[52] Zhu H, Tian P, Zhao J, Zhang H, Wang G, Chen W (2022) A psychobiotic approach to the treatment of depression: a systematic review and meta-analysis. J Funct Foods 91:104999

[53] Dinan TG, Stanton C, Cryan JF (2013) Psychobiotics: a novel class of psychotropic. Biol Psychiat 74(10):720–726 23759244 10.1016/j.biopsych.2013.05.001

[54] Cheng LH, Liu YW, Wu CC, Wang S, Tsai YC (2019) Psychobiotics in mental health, neurodegenerative and neurodevelopmental disorders. J Food Drug Anal 27(3):632–648 31324280 10.1016/j.jfda.2019.01.002 PMC9307042

[55] Tremblay A, Lingrand L, Maillard M, Feuz B, Tompkins TA (2021) The effects of psychobiotics on the microbiota-gut-brain axis in early-life stress and neuropsychiatric disorders. Prog Neuropsychopharmacol Biol Psychiatry 105:110142 33069817 10.1016/j.pnpbp.2020.110142

[56] Lebeer S, Verhoeven TL, Perea Vélez M, Vanderleyden J, De Keersmaecker SC (2007) Impact of environmental and genetic factors on biofilm formation by the probiotic strain Lactobacillus rhamnosus GG. Appl Environ Microbiol 73(21):6768–6775 17827316 10.1128/AEM.01393-07 PMC2074970

[57] Gorbach S, Doron S, Magro F. Lactobacillus rhamnosus GG. InThe microbiota in gastrointestinal pathophysiology 2017 Jan 1 (pp. 79-88). Academic Press

[58] Capurso L (2019) Thirty years of Lactobacillus rhamnosus GG: a review. J Clin Gastroenterol 53:S1–S41 30741841 10.1097/MCG.0000000000001170

[59] Banna GL, Torino F, Marletta F, Santagati M, Salemi R, Cannarozzo E, Falzone L, Ferraù F, Libra M (2017) Lactobacillus rhamnosus GG: an overview to explore the rationale of its use in cancer. Front Pharmacol 1(8):603 10.3389/fphar.2017.00603 PMC5585742 28919861

[60] Alander M, Korpela R, Saxelin M, Vilpponen-Salmela T, Mattila-Sandholm T, Von Wright A (1997) Recovery of Lactobacillus rhamnosus GG from human colonic biopsies. Lett Appl Microbiol 24(5):361–364 9172443 10.1046/j.1472-765x.1997.00140.x

[61] Segers ME, Lebeer S (2014) Towards a better understanding of Lactobacillus rhamnosus GG-host interactions. Microb Cell Fact 13(1):1–16 25186587 10.1186/1475-2859-13-S1-S7 PMC4155824

[62] Tette FM, Kwofie SK, Wilson MD (2022) Therapeutic anti-depressant potential of microbial GABA produced by Lactobacillus rhamnosus strains for GABAergic signaling restoration and inhibition of addiction-induced HPA axis hyperactivity. Curr Issues Mol Biol 44(4):1434–1451 35723354 10.3390/cimb44040096 PMC9164062

[63] Li X, Zheng P, Cao W, Cao Y, She X, Yang H, Ma K, Wu F, Gao X, Fu Y, Yin J (2023) Lactobacillus rhamnosus GG ameliorates noise-induced cognitive deficits and systemic inflammation in rats by modulating the gut-brain axis. Front Cellul Infect Microbiol 26(13):1067367 10.3389/fcimb.2023.1067367 PMC10169735 37180445

[64] Kannampalli P, Pochiraju S, Chichlowski M, Berg BM, Rudolph C, Bruckert M, Miranda A, Sengupta JN (2014) Probiotic L actobacillus rhamnosus GG (LGG) and prebiotic prevent neonatal inflammation-induced visceral hypersensitivity in adult rats. Neurogastroenterol Motility 26(12):1694–704 10.1111/nmo.12450 25298006

[65] Wang L, Zhao R, Li X, Shao P, Xie J, Su X, Xu S, Huang Y, Hu S (2024) Lactobacillus rhamnosus GG improves cognitive impairments in mice with sepsis. PeerJ. 28(12):e17427 10.7717/peerj.17427 PMC11141560 38827289

[66] Xuan H, Umar S, Zhong C, Yu W, Ahmed I, Wheatley JL, Sampath V, Chavez-Bueno S (2024) Lactobacillus rhamnosus modulates murine neonatal gut microbiota and inflammation caused by pathogenic Escherichia coli. BMC Microbiol 24(1):452 39506682 10.1186/s12866-024-03598-6 PMC11539828

[67] Işık M, Özbayer C, Dönmez DB, Erol K, Çolak E, Üstüner MC, Değirmenci İ (2024) Dose-dependent protective effects of Lactobacillus rhamnosus GG against stress-induced ulcer. J Sci Food Agric 104(13):8109–8119 38856115 10.1002/jsfa.13641

[68] Lam EK, Tai EK, Koo MW, Wong HP, Wu WK, Yu L, So WH, Woo PC, Cho CH (2007) Enhancement of gastric mucosal integrity by Lactobacillus rhamnosus GG. Life Sci 80(23):2128–36 17499310 10.1016/j.lfs.2007.03.018

[69] Souza FA, da Silva VG, Bitencourt TB (2017) Use of McFarland Standards and Spectrophotometry for Yarrowia Lipolytica QU69 cell counting. Int J Agric Environ Biotechnol 5:4

[70] Eren I, Nazıroğlu M, Demirdaş A, Çelik Ö, Uğuz AC, Altunbaşak A, Özmen İ, Uz E (2007) Venlafaxine modulates depression-induced oxidative stress in brain and medulla of rat. Neurochem Res 32:497–505 17268845 10.1007/s11064-006-9258-9

[71] Dhir A, Kulkarni SK (2007) Involvement of nitric oxide (NO) signaling pathway in the antidepressant action of bupropion, a dopamine reuptake inhibitor. Eur J Pharmacol 568(1–3):177–185 17509558 10.1016/j.ejphar.2007.04.028

[72] Antoniuk S, Bijata M, Ponimaskin E, Wlodarczyk J (2019) Chronic unpredictable mild stress for modeling depression in rodents: meta-analysis of model reliability. Neurosci Biobehav Rev 99:101–116 30529362 10.1016/j.neubiorev.2018.12.002

[73] Willner P (1984) The validity of animal models of depression. Psychopharmacology 83(1):1–16 6429692 10.1007/BF00427414

[74] Willner P (1990) Animal models of depression: an overview. Pharmacol Ther 45(3):425–455 2405444 10.1016/0163-7258(90)90076-e

[75] Katz RJ, Hersh S (1981) Amitriptyline and scopolamine in an animal model of depression. Neurosci Biobehav Rev 5(2):265–271 7196557 10.1016/0149-7634(81)90008-7

[76] Ding L, Zhang X, Guo H, Yuan J, Li S, Hu W, Golden T, Wu N (2015) The functional study of a Chinese herbal compounded antidepressant medicine–Jie Yu Chu Fan capsule on chronic unpredictable mild stress mouse model. PLoS One. 10(7):e0133405 26186537 10.1371/journal.pone.0133405 PMC4506077

[77] Bessa JM, Mesquita AR, Oliveira M, Pêgo JM, Cerqueira JJ, Palha JA, Almeida OF, Sousa N (2009) A trans-dimensional approach to the behavioral aspects of depression. Front Behavior Neurosci 27(3):352 10.3389/neuro.08.001.2009 PMC2634526 19194528

[78] Strekalova T, Spanagel R, Bartsch D, Henn FA, Gass P (2004) Stress-induced anhedonia in mice is associated with deficits in forced swimming and exploration. Neuropsychopharmacology 29(11):2007–2017 15266352 10.1038/sj.npp.1300532

[79] Liu LL, Li JM, Su WJ, Wang B, Jiang CL (2019) Sex differences in depressive-like behaviour may relate to imbalance of microglia activation in the hippocampus. Brain Behav Immun 81:188–197 31181346 10.1016/j.bbi.2019.06.012

[80] Wang Q, Timberlake MA II, Prall K, Dwivedi Y (2017) The recent progress in animal models of depression. Prog Neuropsychopharmacol Biol Psychiatry 77:99–109 28396255 10.1016/j.pnpbp.2017.04.008 PMC5605906

[81] http://openaccess.ogu.edu.tr:8080/xmlui/bitstream/handle/11684/1940/10289956.pdf?sequence=1&isAllowed=y

[82] Belovicova K, Bogi E, Csatlosova K, Dubovicky M (2017) Animal tests for anxiety-like and depression-like behavior in rats. Interdiscip Toxicol 10(1):40 30123035 10.1515/intox-2017-0006 PMC6096862

[83] Hinojosa FR, Spricigo L Jr, Izídio GS, Brüske GR, Lopes DM, Ramos A (2006) Evaluation of two genetic animal models in behavioral tests of anxiety and depression. Behav Brain Res 168(1):127–136 16324754 10.1016/j.bbr.2005.10.019

[84] Çalışkan H, Fırat AKAT, Zaloğlu N (2017) Şartsız hayvan anksiyete testleri. Ankara Sağlık Hizmetleri Dergisi 16(1):35–40

[85] Rotzinger S, Lovejoy DA, Tan LA (2010) Behavioral effects of neuropeptides in rodent models of depression and anxiety. Peptides 31(4):736–756 20026211 10.1016/j.peptides.2009.12.015

[86] File SE, Seth P (2003) A review of 25 years of the social interaction test. Eur J Pharmacol 463(1–3):35–53 12600701 10.1016/s0014-2999(03)01273-1

[87] Mony TJ, Lee JW, Dreyfus C, DiCicco-Bloom E, Lee HJ (2016) Valproic acid exposure during early postnatal gliogenesis leads to autistic-like behaviors in rats. Clinical Psychopharmacology and Neuroscience 14(4):338 27776385 10.9758/cpn.2016.14.4.338 PMC5083944

[88] Liu X, Yuan J, Guang Y, Wang X, Feng Z (2018) Longitudinal in vivo diffusion tensor imaging detects differential microstructural alterations in the hippocampus of chronic social defeat stress-susceptible and resilient mice. Front Neurosci 12:613 30210285 10.3389/fnins.2018.00613 PMC6123364

[89] Iñiguez SD, Riggs LM, Nieto SJ, Dayrit G, Zamora NN, Shawhan KL, Cruz B, Warren BL (2014) Social defeat stress induces a depression-like phenotype in adolescent male c57BL/6 mice. Stress 17(3):247–55 24689732 10.3109/10253890.2014.910650 PMC5534169

[90] Koç A, Görmüş Zİ (2018) Deney hayvanlarinda anksiyete çalişmalari. Türk Bilimsel Derlemeler Dergisi 11(2):51–7

[91] Küçükkarapinar M, Dönmez A, Candansayar S, Bozkurt A, Akçay E, Gülbahar Ö, Belen HB. Erken Dönem Müdahalelerin Erişkin Wistar Sıçanlarında Davranışsal ve Nörogelişimsel Etkileri.

[92] Cryan JF, Mombereau C (2004) In search of a depressed mouse: utility of models for studying depression-related behavior in genetically modified mice. Mol Psychiatry 9(4):326–357 14743184 10.1038/sj.mp.4001457

[93] Castagné V, Moser P, Roux S, Porsolt RD (2010) Rodent models of depression: forced swim and tail suspension behavioral despair tests in rats and mice. Curr Protoc Pharmacol 49(1):5–8 10.1002/0471141755.ph0508s49 22294373

[94] Porsolt RD, Brossard G, Hautbois C, Roux S (2001) Rodent models of depression: forced swimming and tail suspension behavioral despair tests in rats and mice. Curr Protoc Neurosci 14(1):8–10 10.1002/0471142301.ns0810as14 18428536

[95] Vasconcelos PRCD, Guimarães ABB, Campelo MWS, Vasconcelos PRLD, Guimaraes SB (2015) Preconditioning with L-alanyl-glutamine upon cerebral edema and hypocampus red neurons counting in rats subjected to brain ischemia/reperfusion injury. Acta Cir Bras 30:199–203 25790008 10.1590/S0102-865020150030000006

[96] Mărgăritescu O, Mogoantă L, Pirici I, Pirici D, Cernea D, Mărgăritescu CL (2009) Histopathological changes in acute ischemic stroke. Rom J Morphol Embryol 50(3):327–339 19690757

[97] Pires VLDS, Souza JRFD, Guimarães SB, Silva Filho ARD, Garcia JHP, Vasconcelos PRLD (2011) Preconditioning with L-alanyl-L-glutamine in a Mongolian gerbil model of acute cerebral ischemia/reperfusion injury. Acta Cir Bras 26:14–20 21971651 10.1590/s0102-86502011000700004

[98] Ulloa-Padilla JP, Ghassibi MP, Dubovy SR, Kerr DA (2020) Clinicopathologic correlation of Kaposi sarcoma involving the ocular adnexa: immunophenotyping of diagnostic and therapeutic targets. Ophthalmic Plast Reconstr Surg 36(2):185–190 31743287 10.1097/IOP.0000000000001506

[99] Zhi-han ZH, Dan-yu XU, Guan-yuan CH, Teng TE, Hong-yan WU, Xin-yu ZH. Latest Findings on the Interaction Mechanism Between Depressive Disorder and Intestinal Permeability. Journal of Sichuan University (Medical Science Edition). 2023;54(2). 10.12182/20230360503 PMC10409181 36949682

[100] Marks W, Fournier NM, Kalynchuk LE (2009) Repeated exposure to corticosterone increases depression-like behavior in two different versions of the forced swim test without altering nonspecific locomotor activity or muscle strength. Physiol Behav 98(1–2):67–72 19393673 10.1016/j.physbeh.2009.04.014

[101] Dallman MF, Pecoraro N, Akana SF, La Fleur SE, Gomez F, Houshyar H, Bell ME, Bhatnagar S, Laugero KD, Manalo S (2003) Chronic stress and obesity: a new view of “comfort food.” Proceed Natl Acad Sci 100(20):11696–701 10.1073/pnas.1934666100 PMC208820 12975524

[102] Lu J, Shao RH, Hu L, Tu Y, Guo JY (2016) Potential antiinflammatory effects of acupuncture in a chronic stress model of depression in rats. Neurosci Lett 618:31–38 26921452 10.1016/j.neulet.2016.02.040

[103] Wang D, An SC, Zhang X (2008) Prevention of chronic stress-induced depression-like behavior by inducible nitric oxide inhibitor. Neurosci Lett 433(1):59–64 18248896 10.1016/j.neulet.2007.12.041

[104] Ulker N, Yardimci A, Tektemur NK, Colakoglu N, Ozcan M, Canpolat S, Kelestimur H (2020) Chronic exposure to paroxetine or bupropion modulates the pubertal maturation and the reproductive system in female rats. Reprod Biol 20(2):154–163 32299777 10.1016/j.repbio.2020.03.009

[105] Santamaría A, Arias HR (2010) Neurochemical and behavioral effects elicited by bupropion and diethylpropion in rats. Behav Brain Res 211(1):132–139 20307582 10.1016/j.bbr.2010.03.023

[106] Billes SK, Sinnayah P, Cowley MA (2014) Naltrexone/bupropion for obesity: an investigational combination pharmacotherapy for weight loss. Pharmacol Res 84:1–11 24754973 10.1016/j.phrs.2014.04.004

[107] Wang CH, Gu JY, Zhang XL, Dong J, Yang J, Zhang YL, Ning QF, Shan XW, Li Y (2016) Venlafaxine ameliorates the depression-like behaviors and hippocampal S100B expression in a rat depression model. Behavior Brain Funct 12:1 10.1186/s12993-016-0116-x PMC5146825 27931233

[108] Yilmaz N, Demirdas A, Yilmaz M, Sutcu R, Kirbas A, Cure MC, Eren I (2011) Effects of venlafaxine and escitalopram treatments on NMDA receptors in the rat depression model. J Membr Biol 242:145–151 21755298 10.1007/s00232-011-9385-3

[109] Nie P, Wang M, Zhao Y, Liu S, Chen L, Xu H (2021) Protective effect of Lactobacillus rhamnosus GG on TiO2 nanoparticles-ınduced oxidative stress damage in the liver of young rats. Nanomaterials 11(3):803 33801059 10.3390/nano11030803 PMC8004042

[110] Singh S, Sharma RK, Malhotra S, Pothuraju R, Shandilya UK (2017) Lactobacillus rhamnosus NCDC17 ameliorates type-2 diabetes by improving gut function, oxidative stress and inflammation in high-fat-diet fed and streptozotocintreated rats. Benefic Micro 8(2):243–255 10.3920/BM2016.0090 28008783

[111] Yadav R, Dey DK, Vij R, Meena S, Kapila R, Kapila S (2018) Evaluation of anti-diabetic attributes of Lactobacillus rhamnosus MTCC: 5957, Lactobacillus rhamnosus MTCC: 5897 and Lactobacillus fermentum MTCC: 5898 in streptozotocin induced diabetic rats. Microb Pathog 125:454–462 30316007 10.1016/j.micpath.2018.10.015

[112] Qu N, He Y, Wang C, Xu P, Yang Y, Cai X, Liu H, Yu K, Pei Z, Hyseni I, Sun Z (2020) A POMC-originated circuit regulates stress-induced hypophagia, depression, and anhedonia. Molecul Psychiat 25(5):1006–21 10.1038/s41380-019-0506-1 PMC7056580 31485012

[113] Bruschetta G, Jin S, Liu ZW, Kim JD, Diano S (2020) MC4R signaling in dorsal raphe nucleus controls feeding, anxiety, and depression. Cell Rep 33(2):108267 33053350 10.1016/j.celrep.2020.108267

[114] Chaffin AT, Fang Y, Larson KR, Mul JD, Ryan KK (2019) Sex-dependent effects of MC4R genotype on HPA axis tone: implications for stress-associated cardiometabolic disease. Stress 22(5):571–580 31184537 10.1080/10253890.2019.1610742 PMC6690797

[115] Lim BK, Huang KW, Grueter BA, Rothwell PE, Malenka RC (2012) Anhedonia requires MC4R-mediated synaptic adaptations in nucleus accumbens. Nature 487(7406):183–189 22785313 10.1038/nature11160 PMC3397405

[116] Liu MY, Yin CY, Zhu LJ, Zhu XH, Xu C, Luo CX, Chen H, Zhu DY, Zhou QG (2018) Sucrose preference test for measurement of stress-induced anhedonia in mice. Nature Protocol 13(7):1686–98 10.1038/s41596-018-0011-z 29988104

[117] Markov DD (2022) Sucrose preference test as a measure of anhedonic behavior in a chronic unpredictable mild stress model of depression: outstanding issues. Brain Sci 12(10):1287 36291221 10.3390/brainsci12101287 PMC9599556

[118] Powell TR, Fernandes C, Schalkwyk LC (2012) Depression-related behavioral tests. Curr Protocol Mouse Biol 2(2):119–127 10.1002/9780470942390.mo110176 26069008

[119] Hong S, Flashner B, Chiu M, ver Hoeve E, Luz S, Bhatnagar S. Social isolation in adolescence alters behaviors in the forced swim and sucrose preference tests in female but not in male rats. Physiology & behavior. 2012 Jan 18;105(2):269-75. 10.1016/j.physbeh.2011.08.036 PMC3260053 21907226

[120] Sáenz JCB, Villagra OR, Trías JF (2006) Factor analysis of forced swimming test, sucrose preference test and open field test on enriched, social and isolated reared rats. Behav Brain Res 169(1):57–65 16414129 10.1016/j.bbr.2005.12.001

[121] Wang J, Xu S, Chen X, Wang L, Li J, Li G, Zhang B (2020) Antidepressant effect of EGCG through the inhibition of hippocampal neuroinflammation in chronic unpredictable mild stress-induced depression rat model. J Funct Foods 73:104106

[122] Araghi A, Abbasabadi BM, Talebpour N, Golshahi H. The Chronic Administration of Persian Herbal Formulas Psycodigest Induced The Antidepressant-Like Effect in a Chronic Unpredictable Mild Stress (CUMS) Mouse Model.

[123] Zorkina YA, Zubkov EA, Morozova AY, Ushakova VM, Chekhonin VP (2019) The comparison of a new ultrasound-induced depression model to the chronic mild stress paradigm. Front Behav Neurosci 13:146 31312126 10.3389/fnbeh.2019.00146 PMC6614435

[124] Abo-youssef AM (2016) Possible antidepressant effects of vanillin against experimentally induced chronic mild stress in rats. Beni-Suef Univ J Basic Appl Sci 5(2):187–192

[125] Xie R, Jiang P, Lin LI, Jiang J, Yu B, Rao J, Liu H, Wei W, Qiao YI (2020) Oral treatment with Lactobacillus reuteri attenuates depressive-like behaviors and serotonin metabolism alterations induced by chronic social defeat stress. J Psychiatric Res 1(122):70–8 10.1016/j.jpsychires.2019.12.013 31927268

[126] Liang S, Wang T, Hu X, Luo J, Li W, Wu X, Duan Y, Jin F (2015) Administration of Lactobacillus helveticus NS8 improves behavioral, cognitive, and biochemical aberrations caused by chronic restraint stress. Neuroscience 3(310):561–77 10.1016/j.neuroscience.2015.09.033 26408987

[127] Ho YJ, Eichendorff J, Schwarting RK (2002) Individual response profiles of male Wistar rats in animal models for anxiety and depression. Behav Brain Res 136(1):1–12 12385785 10.1016/s0166-4328(02)00089-x

[128] Kompagne H, Bárdos G, Szénási G, Gacsályi I, Hársing LG, Lévay G (2008) Chronic mild stress generates clear depressive but ambiguous anxiety-like behaviour in rats. Behav Brain Res 193(2):311–314 18590771 10.1016/j.bbr.2008.06.008

[129] Carrasco MC, Vicens P, Vidal J, Redolat R (2004) Effects of acute administration of bupropion on behavior in the elevated plus-maze test by NMRI mice. Prog Neuropsychopharmacol Biol Psychiatry 28(7):1135–1141 15610926 10.1016/j.pnpbp.2004.06.005

[130] Redolat R, Gómez MC, Vicens P, Carrasco MC (2005) Bupropion effects on aggressiveness and anxiety in OF1 male mice. Psychopharmacology 177:418–427 15289998 10.1007/s00213-004-1965-9

[131] Lapmanee S, Charoenphandhu J, Teerapornpuntakit J, Krishnamra N, Charoenphandhu N (2017) Agomelatine, venlafaxine, and running exercise effectively prevent anxiety-and depression-like behaviors and memory impairment in restraint stressed rats. PLoS ONE 12(11):e0187671 29099859 10.1371/journal.pone.0187671 PMC5669450

[132] Darwish IE, Maklad HM, Diab IH (2013) Behavioral and neuronal biochemical possible effects in experimental induced chronic mild stress in male albino rats under the effect of oral barley administration in comparison to venlafaxine. Int J Physiol, Pathophysiol Pharmacol 5(2):128 23750311 PMC3669741

[133] Liu Y, Mian MF, Neufeld KAM, Forsythe P (2020) CD4+ CD25+ T cells are essential for behavioral effects of Lactobacillus rhamnosus JB-1 in male BALB/c mice. Brain Behav Immun 88:451–460 32276029 10.1016/j.bbi.2020.04.014

[134] Lai CT, Chen CY, She SC, Chen WJ, Kuo TB, Lin HC, Yang CC (2022) Production of Lactobacillus brevis ProGA28 attenuates stress-related sleep disturbance and modulates the autonomic nervous system and the motor response in anxiety/depression behavioral tests in Wistar-Kyoto rats. Life Sci 288:120165 34822793 10.1016/j.lfs.2021.120165

[135] Can A, Dao D T, Arad M, Terrillion CE, Piantadosi SC, Gould TD The mouse forced swim test. JoVE (Journal of Visualized Experiments), (59), e3638 (2012) 10.3791/3638 PMC3353513 22314943

[136] O’Neil MF, Moore NA (2003) Animal models of depression: are there any? Hum Psychopharmacol Clin Exp 18(4):239–254 10.1002/hup.496 12766928

[137] Abelaira HM, Réus GZ, Quevedo J (2013) Animal models as tools to study the pathophysiology of depression. Brazilian J Psychiat 35:S112–S120 10.1590/1516-4446-2013-1098 24271223

[138] Petit-Demouliere B, Chenu F, Bourin M (2005) Forced swimming test in mice: a review of antidepressant activity. Psychopharmacology 177:245–255 15609067 10.1007/s00213-004-2048-7

[139] Ye YL, Zhong K, Liu DD, Xu J, Pan BB, Li X, Yu YP, Zhang Q (2017) Huanglian-Jie-Du-Tang extract ameliorates depression-like behaviors through BDNF-TrkB-CREB pathway in rats with chronic unpredictable stress. Evidence-Based Complement Alternat Med 2017(1):7903918 10.1155/2017/7903918 PMC5488320 28694833

[140] Abdel-Wahab BA, Salama RH (2011) Venlafaxine protects against stress-induced oxidative DNA damage in hippocampus during antidepressant testing in mice. Pharmacol Biochem Behav 100(1):59–65 21835191 10.1016/j.pbb.2011.07.015

[141] Faucher P, Dries A, Mousset PY, Leboyer M, Dore J, Beracochea D (2022) Synergistic effects of Lacticaseibacillus rhamnosus GG, glutamine, and curcumin on chronic unpredictable mild stress-induced depression in a mouse model. Beneficial Microbes 13(3):253–264 35786408 10.3920/BM2021.0188

[142] Overstreet DH, Commissaris RC, De La Garza II, File SE, Knapp DJ, Seiden LS. Involvement of 5-HT 1A Receptors in Animal Tests of Anxiety and Depression: Evidence from Genetic Models. Stress: The International Journal on the Biology of Stress. 2003 May 1;6(2). 10.1080/1025389031000111311 12775329

[143] Paolo SD, Brain P, Willner P (1994) Effects of chronic mild stress on performance in behavioural tests relevant to anxiety and depression. Physiol Behav 56(5):861–867 7824585 10.1016/0031-9384(94)90316-6

[144] Saavedra-Rodríguez L, Feig LA (2013) Chronic social instability induces anxiety and defective social interactions across generations. Biol Psychiat 73(1):44–53 22906514 10.1016/j.biopsych.2012.06.035 PMC3826464

[145] Lipina TV, Fletcher PJ, Lee FH, Wong AH, Roder JC (2013) Disrupted-in-schizophrenia-1 Gln31Leu polymorphism results in social anhedonia associated with monoaminergic imbalance and reduction of CREB and β-arrestin-1, 2 in the nucleus accumbens in a mouse model of depression. Neuropsychopharmacology 38(3):423–436 23011268 10.1038/npp.2012.197 PMC3547193

[146] Mori T, Shibasaki M, Ogawa Y, Hokazono M, Wang TC, Rahmadi M, Suzuki T (2013) Comparison of the behavioral effects of bupropion and psychostimulants. Eur J Pharmacol 718(1–3):370–375 23993950 10.1016/j.ejphar.2013.07.046

[147] Yang CH, Shi HS, Zhu WL, Wu P, Sun LL, Si JJ, Liu MM, Zhang Y, Suo L, Yang JL (2012) Venlafaxine facilitates between-session extinction and prevents reinstatement of auditory-cue conditioned fear. Behav Brain Res 230(1):268–73 22366271 10.1016/j.bbr.2012.02.023

[148] Venzala E, García-García AL, Elizalde N, Delagrange P, Tordera RM (2012) Chronic social defeat stress model: behavioral features, antidepressant action, and interaction with biological risk factors. Psychopharmacology 224:313–325 22707231 10.1007/s00213-012-2754-5

[149] Gray VC, Hughes RN (2015) Drug-, dose-and sex-dependent effects of chronic fluoxetine, reboxetine and venlafaxine on open-field behavior and spatial memory in rats. Behav Brain Res 281:43–54 25523028 10.1016/j.bbr.2014.12.023

[150] Kong Q, Wang B, Tian P, Li X, Zhao J, Zhang H, Chen W, Wang G (2021) Daily intake of Lactobacillus alleviates autistic-like behaviors by ameliorating the 5-hydroxytryptamine metabolic disorder in VPA-treated rats during weaning and sexual maturation. Food Funct 12(6):2591–604 33629689 10.1039/d0fo02375b

[151] Arseneault-Breard J, Rondeau I, Gilbert K, Girard SA, Tompkins TA, Godbout R, Rousseau G (2012) Combination of Lactobacillus helveticus R0052 and Bifidobacterium longum R0175 reduces post-myocardial infarction depression symptoms and restores intestinal permeability in a rat model. Br J Nutr 107(12):1793–1799 21933458 10.1017/S0007114511005137

[152] Murakami S, Imbe H, Morikawa Y, Kubo C, Senba E (2005) Chronic stress, as well as acute stress, reduces BDNF mRNA expression in the rat hippocampus but less robustly. Neurosci Res 53(2):129–139 16024125 10.1016/j.neures.2005.06.008

[153] Marmigère F, Givalois L, Rage F, Arancibia S, Tapia-Arancibia L (2003) Rapid induction of BDNF expression in the hippocampus during immobilization stress challenge in adult rats. Hippocampus 13(5):646–655 12921353 10.1002/hipo.10109

[154] Liu Z, Qi Y, Cheng Z, Zhu X, Fan C, Yu SY (2016) The effects of ginsenoside Rg1 on chronic stress induced depression-like behaviors, BDNF expression and the phosphorylation of PKA and CREB in rats. Neuroscience 322:358–369 26926964 10.1016/j.neuroscience.2016.02.050

[155] Jiang Y, Zhu J (2015) Effects of sleep deprivation on behaviors and abnormal hippocampal BDNF/miR-10B expression in rats with chronic stress depression. Int J Clin Exp Pathol 8(1):586 25755749 PMC4348910

[156] Torregrossa MM, Folk JE, Rice KC, Watson SJ, Woods JH (2005) Chronic administration of the delta opioid receptor agonist (+) BW373U86 and antidepressants on behavior in the forced swim test and BDNF mRNA expression in rats. Psychopharmacology 183:31–40 16220339 10.1007/s00213-005-0113-5 PMC1315298

[157] Gao C, Kong S, Guo B, Liang X, Duan H, Li D (2019) Antidepressive effects of Taraxacum officinale in a mouse model of depression are due to inhibition of corticosterone levels and modulation of mitogen-activated protein kinase phosphatase-1 (mkp-1) and brain-derived neurotrophic factor (bdnf) expression. Med Sci Monit: Int Med J Experiment Clin Res 25:389 10.12659/MSM.912922 PMC6340315 30636257

[158] Kondo S, El Omri A, Han J, Isoda H (2015) Antidepressant-like effects of rosmarinic acid through mitogen-activated protein kinase phosphatase-1 and brain-derived neurotrophic factor modulation. J Funct Foods 14:758–766

[159] Li JJ, Yuan YG, Hou G, Zhang XR (2011) Dose-related effects of venlafaxine on pCREB and brain-derived neurotrophic factor (BDNF) in the hippocampus of the rat by chronic unpredictable stress. Acta Neuropsychiatrica 23(1):20–30 25379693 10.1111/j.1601-5215.2010.00512.x

[160] Cheng R, Xu T, Zhang Y, Wang F, Zhao L, Jiang Y, He F (2020) Lactobacillus rhamnosus GG and Bifidobacterium bifidum TMC3115 can affect development of hippocampal neurons cultured in vitro in a strain-dependent manner. Probiot Antimicro Prot 12:589–599 10.1007/s12602-019-09571-4 31286435

[161] Oh NS, Joung JY, Lee JY, Song JG, Oh S, Kim Y, Kim HW, Kim SH (2020) Glycated milk protein fermented with Lactobacillus rhamnosus ameliorates the cognitive health of mice under mild-stress condition. Gut Microbes 11(6):1643–61 32573326 10.1080/19490976.2020.1756690 PMC7524334

[162] Kim MH, Leem YH (2014) Chronic exercise improves repeated restraint stress-induced anxiety and depression through 5HT1A receptor and cAMP signaling in hippocampus. J Exerc Nutr Biochem 18(1):97 10.5717/jenb.2014.18.1.97 PMC4241932 25566444

[163] Moret C, Briley M (2011) The importance of norepinephrine in depression. Neuropsychiatr Dis Treat 7(sup1):9–13 21750623 10.2147/NDT.S19619 PMC3131098

[164] Jovanovic H, Perski A, Berglund H, Savic I (2011) Chronic stress is linked to 5-HT1A receptor changes and functional disintegration of the limbic networks. Neuroimage 55(3):1178–1188 21211567 10.1016/j.neuroimage.2010.12.060

[165] López JF, Chalmers DT, Little KY, Watson SJ (1998) Regulation of serotonin1A, glucocorticoid, and mineralocorticoid receptor in rat and human hippocampus: implications for the neurobiology of depression. Biol Psychiat 43(8):547–573 9564441 10.1016/s0006-3223(97)00484-8

[166] Van Riel E, Meijer OC, Steenbergen PJ, Joels M (2003) Chronic unpredictable stress causes attenuation of serotonin responses in cornu ammonis 1 pyramidal neurons. Neuroscience 120(3):649–658 12895506 10.1016/s0306-4522(03)00355-5

[167] Guiard BP, Mansari ME, Blier P (2009) Prospect of a dopamine contribution in the next generation of antidepressant drugs: the triple reuptake inhibitors. Curr Drug Targets 10(11):1069–1084 19702555 10.2174/138945009789735156

[168] Mansari ME, Manta S, Oosterhof C, El Iskandrani KS, Chenu F, Shim S, Blier P. Restoration of serotonin neuronal firing following long-term administration of bupropion but not paroxetine in olfactory bulbectomized rats. International Journal of Neuropsychopharmacology. 2015 Feb 1;18(4):pyu050. 10.1093/ijnp/pyu050 PMC4360219 25522394

[169] Fenli S, Feng W, Ronghua Z, Huande L (2013) Biochemical mechanism studies of venlafaxine by metabonomic method in rat model of depression. Eur Rev Med Pharmacol Sci 17(1):41–48 23329522

[170] Cheng LH, Chou PY, Hou AT, Huang CL, Shiu WL, Wang S (2022) Lactobacillus paracasei PS23 improves cognitive deficits via modulating the hippocampal gene expression and the gut microbiota in D-galactose-induced aging mice. Food Funct 13(9):5240–5251 35438699 10.1039/d2fo00165a

[171] Leggio GM, Salomone S, Bucolo C, Platania C, Micale V, Caraci F, Drago F (2013) Dopamine D3 receptor as a new pharmacological target for the treatment of depression. Eur J Pharmacol 719(1–3):25–33 23872400 10.1016/j.ejphar.2013.07.022

[172] Sim HR, Choi TY, Lee HJ, Kang EY, Yoon S, Han PL, Choi SY, Baik JH (2013) Role of dopamine D2 receptors in plasticity of stress-induced addictive behaviours. Nature Commun 4(1):1579 23481387 10.1038/ncomms2598

[173] Du Y, Gao XR, Peng L, Ge JF (2020) Crosstalk between the microbiota-gut-brain axis and depression. Heliyon 6(6):e04097 32529075 10.1016/j.heliyon.2020.e04097 PMC7276434

[174] Xia QP, Cheng ZY, He L (2019) The modulatory role of dopamine receptors in brain neuroinflammation. Int Immunopharmacol 76:105908 31622861 10.1016/j.intimp.2019.105908

[175] Wang T, Nowrangi D, Yu L, Lu T, Tang J, Han B, Ding Y, Fu F, Zhang JH (2018) Activation of dopamine D1 receptor decreased NLRP3-mediated inflammation in intracerebral hemorrhage mice. J Neuroinflamm 15:1 10.1186/s12974-017-1039-7 PMC5753458 29301581

[176] Gorwood P, Le Strat Y, Ramoz N, Dubertret C, Moalic JM, Simonneau M (2012) Genetics of dopamine receptors and drug addiction. Hum Genet 131:803–822 22350797 10.1007/s00439-012-1145-7

[177] Cao G, Meng G, Zhu L, Zhu J, Dong N, Zhou X, Zhang S, Zhang Y (2021) Susceptibility to chronic immobilization stress-induced depressive-like behaviour in middle-aged female mice and accompanying changes in dopamine D1 and GABA A receptors in related brain regions. Behav Brain Funct 17:1–9 33863350 10.1186/s12993-021-00175-z PMC8052654

[178] Yanovich C, Kirby ML, Michaelevski I, Yadid G, Pinhasov A (2018) Social rank-associated stress vulnerability predisposes individuals to cocaine attraction. Sci Rep 8(1):1759 29379100 10.1038/s41598-018-19816-x PMC5789078

[179] Sabti M, Sasaki K, Gadhi C, Isoda H. Elucidation of the molecular mechanism underlying Lippia citriodora (Lim.)-induced relaxation and anti-depression. International journal of molecular sciences. 2019 Jul 20;20(14):3556. 10.3390/ijms20143556 PMC6678442 31330819

[180] Shuto T, Kuroiwa M, Sotogaku N, Kawahara Y, Oh YS, Jang JH, Shin CH, Ohnishi YN, Hanada Y, Miyakawa T, Kim Y (2020) Obligatory roles of dopamine D1 receptors in the dentate gyrus in antidepressant actions of a selective serotonin reuptake inhibitor, fluoxetine. Molecular Psychiat 25(6):1229–44 10.1038/s41380-018-0316-x PMC7244404 30531938

[181] Hudson AL, Lalies MD, Silverstone P (2012) Venlafaxine enhances the effect of bupropion on extracellular dopamine in rat frontal cortex. Can J Physiol Pharmacol 90(6):803–809 22512539 10.1139/y2012-045

[182] Mineur YS, Cahuzac EL, Mose TN, Bentham MP, Plantenga ME, Thompson DC, Picciotto MR (2018) Interaction between noradrenergic and cholinergic signaling in amygdala regulates anxiety-and depression-related behaviors in mice. Neuropsychopharmacology 43(10):2118–2125 29472646 10.1038/s41386-018-0024-x PMC6098039

[183] Madison DV, Nicoll RA (1988) Norepinephrine decreases synaptic inhibition in the rat hippocampus. Brain Res 442(1):131–138 2834010 10.1016/0006-8993(88)91440-0

[184] Ressler KJ, Nemeroff CB (1999) Role of norepinephrine in the pathophysiology and treatment of mood disorders. Biol Psychiat 46(9):1219–1233 10560027 10.1016/s0006-3223(99)00127-4

[185] Drouin C, Bobadilla AC, Tassin JP (2017) Norepinephrine. Reference module in neuroscience and biobehavioral psychology

[186] Shishkina GT, Kalinina TS, Dygalo NN (2004) Attenuation of α2A-adrenergic receptor expression in neonatal rat brain by RNA interference or antisense oligonucleotide reduced anxiety in adulthood. Neuroscience 129(3):521–528 15541874 10.1016/j.neuroscience.2004.08.015

[187] Cottingham C, Wang Q (2012) α2 adrenergic receptor dysregulation in depressive disorders: ımplications for the neurobiology of depression and antidepressant therapy. Neurosci Biobehav Rev 36(10):2214–2225 22910678 10.1016/j.neubiorev.2012.07.011 PMC3508310

[188] Wakeno M, Kato M, Okugawa G, Fukuda T, Hosoi Y, Takekita Y, Yamashita M, Nonen S, Azuma J, Kinoshita T (2008) The alpha 2A-adrenergic receptor gene polymorphism modifies antidepressant responses to milnacipran. J Clin Psychopharmacol 28(5):518–24 18794646 10.1097/JCP.0b013e31818455fc

[189] Galyamina AG, Kovalenko IL, Smagin DA, Kudryavtseva NN (2017) Altered expression of neurotransmitters systems’ genes in the ventral tegmental area of depressive male mice: data of RNA-Seq. Zhurnal Vysshei Nervnoi Deiatelnosti Imeni IP Pavlova 67(1):113–128 30695556

[190] Du Y, Ruan J, Zhang L, Fu F (2020) Jieyu anshen granule, a Chinese herbal formulation, exerts effects on poststroke depression in rats. Evid-Based Complement Alternat Med 2020(1):7469068 32184899 10.1155/2020/7469068 PMC7060433

[191] Ghanbari R, El Mansari M, Blier P (2011) Enhancement of serotonergic and noradrenergic neurotransmission in the rat hippocampus by sustained administration of bupropion. Psychopharmacology 217:61–73 21445565 10.1007/s00213-011-2260-1

[192] Estévez-Cabrera MM, Sánchez-Muñoz F, Pérez-Sánchez G, Pavón L, Hernández-Díazcouder A, Altamirano JL, Soria-Fregoso C, Alfaro-Rodríguez A, Bonilla-Jaime H. Therapeutic treatment with fluoxetine using the chronic unpredictable stress model induces changes in neurotransmitters and circulating miRNAs in extracellular vesicles. Heliyon. 2023 Feb 1;9(2). 10.1016/j.heliyon.2023.e13442 PMC9958461 36852042

[193] Page ME, Abercrombie ED (1997) An analysis of the effects of acute and chronic fluoxetine on extracellular norepinephrine in the rat hippocampus during stress. Neuropsychopharmacology 16(6):419–425 9165497 10.1016/S0893-133X(96)00281-3

[194] Liao JF, Cheng YF, You ST, Kuo WC, Huang CW, Chiou JJ, Hsu CC, Hsieh-Li HM, Wang S, Tsai YC (2020) Lactobacillus plantarum PS128 alleviates neurodegenerative progression in 1-methyl-4-phenyl-1, 2, 3, 6-tetrahydropyridine-induced mouse models of Parkinson’s disease. Brain Behav İmmun 1(90):26–46 10.1016/j.bbi.2020.07.036 32739365

[195] Duman RS, Sanacora G, Krystal JH (2019) Altered connectivity in depression: GABA and glutamate neurotransmitter deficits and reversal by novel treatments. Neuron 102(1):75–90 30946828 10.1016/j.neuron.2019.03.013 PMC6450409

[196] Fogaça MV, Duman RS (2019) Cortical GABAergic dysfunction in stress and depression: new insights for therapeutic interventions. Front Cellul Neurosci 12(13):448587 10.3389/fncel.2019.00087 PMC6422907 30914923

[197] Holm MM, Nieto-Gonzalez JL, Vardya I, Henningsen K, Jayatissa MN, Wiborg O, Jensen K (2011) Hippocampal GABAergic dysfunction in a rat chronic mild stress model of depression. Hippocampus 21(4):422–433 20087886 10.1002/hipo.20758

[198] Skilbeck KJ, Johnston GA, Hinton T (2010) Stress and GABAA receptors. J Neurochem 112(5):1115–1130 20002524 10.1111/j.1471-4159.2009.06539.x

[199] Skórzewska A, Lehner M, Wisłowska-Stanek A, Krząścik P, Ziemba A, Płaźnik A (2014) The effect of chronic administration of corticosterone on anxiety-and depression-like behavior and the expression of GABA-A receptor alpha-2 subunits in brain structures of low-and high-anxiety rats. Horm Behav 65(1):6–13 24200620 10.1016/j.yhbeh.2013.10.011

[200] Jacobson-Pick S, Richter-Levin G (2012) Short-and long-term effects of juvenile stressor exposure on the expression of GABAA receptor subunits in rats. Stress 15(4):416–424 22044189 10.3109/10253890.2011.634036

[201] Montpied P, Weizman A, Weizman R, Kook KA, Morrow AL, Paul SM (1993) Repeated swim-stress reduces GABAA receptor α subunit mRNAs in the mouse hippocampus. Mol Brain Res 18(3):267–272 7684486 10.1016/0169-328x(93)90199-y

[202] Zheng G, Zhang X, Chen Y, Zhang Y, Luo W, Chen J (2007) Evidence for a role of GABAA receptor in the acute restraint stress-induced enhancement of spatial memory. Brain Res 1181:61–73 17916335 10.1016/j.brainres.2007.08.077

[203] Martisova E, Solas M, Horrillo I, Ortega JE, Meana JJ, Tordera RM, Ramírez MJ (2012) Long lasting effects of early-life stress on glutamatergic/GABAergic circuitry in the rat hippocampus. Neuropharmacology 62(5–6):1944–1953 22245561 10.1016/j.neuropharm.2011.12.019

[204] McVey Neufeld KA, O’Mahony SM, Hoban AE, Waworuntu RV, Berg BM, Dinan TG, Cryan JF (2019) Neurobehavioural effects of Lactobacillus rhamnosus GG alone and in combination with prebiotics polydextrose and galactooligosaccharide in male rats exposed to early-life stress. Nutr Neurosci 22(6):425–434 29173065 10.1080/1028415X.2017.1397875

[205] Rodríguez-Gaztelumendi A, Rojo ML, Pazos A, Díaz A (2009) Altered CB1 receptor-signaling in prefrontal cortex from an animal model of depression is reversed by chronic fluoxetine. J Neurochem 108(6):1423–1433 19183263 10.1111/j.1471-4159.2009.05898.x

[206] Ruehle S, Remmers F, Romo-Parra H, Massa F, Wickert M, Wörtge S, Häring M, Kaiser N, Marsicano G, Pape HC, Lutz B (2013) Cannabinoid CB1 receptor in dorsal telencephalic glutamatergic neurons: distinctive sufficiency for hippocampus-dependent and amygdala-dependent synaptic and behavioral functions. J Neurosci 33(25):10264–77 23785142 10.1523/JNEUROSCI.4171-12.2013 PMC6618598

[207] Davies SN, Pertwee RG, Riedel G (2002) Functions of cannabinoid receptors in the hippocampus. Neuropharmacology 42(8):993–1007 12128000 10.1016/s0028-3908(02)00060-6

[208] Kirilly E, Gonda X, Bagdy G (2012) CB 1 receptor antagonists: new discoveries leading to new perspectives. Acta Physiol 205(1):41–60 10.1111/j.1748-1716.2012.02402.x 22463610

[209] McLaughlin RJ, Hill MN, Morrish AC, Gorzalka BB (2007) Local enhancement of cannabinoid CB1 receptor signalling in the dorsal hippocampus elicits an antidepressant-like effect. Behav Pharmacol 18(5–6):431–438 17762511 10.1097/FBP.0b013e3282ee7b44

[210] Kirkedal C, Elfving B, Müller HK, Moreira FA, Bindila L, Lutz B, Wegener G, Liebenberg N (2019) Hemisphere-dependent endocannabinoid system activity in prefrontal cortex and hippocampus of the Flinders Sensitive Line rodent model of depression. Neurochem Int 1(125):7–15 10.1016/j.neuint.2019.01.023 30716357

[211] Segev A, Rubin AS, Abush H, Richter-Levin G, Akirav I (2014) Cannabinoid receptor activation prevents the effects of chronic mild stress on emotional learning and LTP in a rat model of depression. Neuropsychopharmacology 39(4):919–933 24141570 10.1038/npp.2013.292 PMC3924526

[212] Ballesta A, Orio L, Arco R, Vargas A, Romero-Sanchiz P, Nogueira-Arjona R, de Heras RG, Antón M, Ramírez-López M, Serrano A, Pavón FJ (2019) Bupropion, a possible antidepressant without negative effects on alcohol relapse. Eur Neuropsychopharmacol 29(6):756–65 31064683 10.1016/j.euroneuro.2019.03.012

[213] Salort G, Hernández-Hernández E, García-Fuster MJ, García-Sevilla JA (2020) Regulation of cannabinoid CB1 and CB2 receptors, neuroprotective mTOR and pro-apoptotic JNK1/2 kinases in postmortem prefrontal cortex of subjects with major depressive disorder. J Affect Disord 276:626–635 32871695 10.1016/j.jad.2020.07.074

[214] Gioacchini G, Rossi G, Carnevali O (2017) Host-probiotic interaction: new insight into the role of the endocannabinoid system by in vivo and ex vivo approaches. Sci Rep 7(1):1261 28455493 10.1038/s41598-017-01322-1 PMC5430882

[215] Juruena MF (2014) Early-life stress and HPA axis trigger recurrent adulthood depression. Epilepsy Behav 38:148–159 24269030 10.1016/j.yebeh.2013.10.020

[216] De Kloet ER, Van Acker SA, Sibug RM, Oitzl MS, Meijer OC, Rahmouni K, De Jong W (2000) Brain mineralocorticoid receptors and centrally regulated functions. Kidney Int 57(4):1329–1336 10760063 10.1046/j.1523-1755.2000.00971.x

[217] Wang H, Meyer K, Korz V (2013) Stress induced hippocampal mineralocorticoid and estrogen receptor β gene expression and long-term potentiation in male adult rats is sensitive to early-life stress experience. Psychoneuroendocrinology 38(2):250–262 22776422 10.1016/j.psyneuen.2012.06.004

[218] Zhe D, Fang H, Yuxiu S (2008) Expressions of hippocampal mineralocorticoid receptor (MR) and glucocorticoid receptor (GR) in the single-prolonged stress-rats. Acta Histochem Cytochem 41(4):89–95 18787639 10.1267/ahc.08013 PMC2532603

[219] Kitamura Y, Fujitani Y, Kitagawa K, Miyazaki T, Sagara H, Kawasaki H, Shibata K, Sendo T, Gomita Y (2008) Effects of imipramine and bupropion on the duration of immobility of ACTH-treated rats in the forced swim test: involvement of the expression of 5-HT2A receptor mRNA. Biol Pharmaceut Bullet 31(2):246–9 10.1248/bpb.31.246 18239281

[220] Liu M, Song S, Hu C, Tang L, Lam JC, Lam PK, Chen L (2020) Dietary administration of probiotic Lactobacillus rhamnosus modulates the neurological toxicities of perfluorobutanesulfonate in zebrafish. Environ Pollut 265:114832 32454362 10.1016/j.envpol.2020.114832

[221] Coll RC, O’Neill LAJ, Schroder K (2016) Questions and controversies in innate immune research: what is the physiological role of NLRP3? Cell death discovery 2(1):1–5 10.1038/cddiscovery.2016.19 PMC4979470 27551512

[222] Kaufmann FN, Costa AP, Ghisleni G, Diaz AP, Rodrigues ALS, Peluffo H, Kaster MP (2017) NLRP3 inflammasome-driven pathways in depression: clinical and preclinical findings. Brain Behav Immun 64:367–383 28263786 10.1016/j.bbi.2017.03.002

[223] Pellegrini C, Martelli A, Antonioli L, Fornai M, Blandizzi C, Calderone V (2021) NLRP3 inflammasome in cardiovascular diseases: pathophysiological and pharmacological implications. Med Res Rev 41(4):1890–1926 33460162 10.1002/med.21781

[224] Jiang P, Guo Y, Dang R, Yang M, Liao D, Li H, Sun Z, Feng Q, Xu P (2017) Salvianolic acid B protects against lipopolysaccharide-induced behavioral deficits and neuroinflammatory response: involvement of autophagy and NLRP3 inflammasome. J Neuroinflamm 14:1 10.1186/s12974-017-1013-4 PMC5719935 29212498

[225] Glick D, Barth S, Macleod KF (2010) Autophagy: cellular and molecular mechanisms. J Pathol 221(1):3–12 20225336 10.1002/path.2697 PMC2990190

[226] Biasizzo M, Kopitar-Jerala N (2020) Interplay between NLRP3 inflammasome and autophagy. Front Immunol 11:591803 33163006 10.3389/fimmu.2020.591803 PMC7583715

[227] Cao Z, Wang Y, Long Z, He G (2019) Interaction between autophagy and the NLRP3 inflammasome. Acta Biochim Biophys Sin 51(11):1087–1095 31609412 10.1093/abbs/gmz098

[228] Li R, Wang X, Qin T, Qu R, Ma S (2016) Apigenin ameliorates chronic mild stress-induced depressive behavior by inhibiting interleukin-1β production and NLRP3 inflammasome activation in the rat brain. Behav Brain Res 296:318–325 26416673 10.1016/j.bbr.2015.09.031

[229] Yue N, Huang H, Zhu X, Han Q, Wang Y, Li B, Liu Q, Wu G, Zhang Y, Yu J (2017) Activation of P2X7 receptor and NLRP3 inflammasome assembly in hippocampal glial cells mediates chronic stress-induced depressive-like behaviors. J Neuroinflamm 14:1–5 10.1186/s12974-017-0865-y PMC5424302 28486969

[230] Brustolim D, Ribeiro-dos-Santos R, Kast RE, Altschuler EL, Soares MBP (2006) A new chapter opens in anti-inflammatory treatments: the antidepressant bupropion lowers production of tumor necrosis factor-alpha and interferon-gamma in mice. Int Immunopharmacol 6(6):903–907 16644475 10.1016/j.intimp.2005.12.007

[231] Trojan E, Chamera K, Bryniarska N, Kotarska K, Leśkiewicz M, Regulska M, Basta-Kaim A (2019) Role of chronic administration of antidepressant drugs in the prenatal stress-evoked inflammatory response in the brain of adult offspring rats: involvement of the nlrp3 inflammasome-related pathway. Mol Neurobiol 56:5365–5380 30610610 10.1007/s12035-018-1458-1 PMC6614144

[232] Wu Q, Liu MC, Yang J, Wang JF, Zhu YH (2016) Lactobacillus rhamnosus GR-1 ameliorates Escherichia coli-induced inflammation and cell damage via attenuation of ASC-independent NLRP3 inflammasome activation. Appl Environ Microbiol 82(4):1173–1182 26655757 10.1128/AEM.03044-15 PMC4751844

[233] Miettinen M, Pietilä TE, Kekkonen RA, Kankainen M, Latvala S, Pirhonen J, Österlund P, Korpela R, Julkunen I (2012) Nonpathogenic Lactobacillus rhamnosus activates the inflammasome and antiviral responses in human macrophages. Gut Microbes 3(6):510–22 22895087 10.4161/gmic.21736 PMC3495788

[234] Fernández-García V, González-Ramos S, Martín-Sanz P, Garcia-del Portillo F, Laparra JM, Boscá L (2021) NOD1 in the interplay between microbiota and gastrointestinal immune adaptations. Pharmacol Res 171:105775 34273489 10.1016/j.phrs.2021.105775

[235] Masumoto J, Yang K, Varambally S, Hasegawa M, Tomlins SA, Qiu S, Fujimoto Y, Kawasaki A, Foster SJ, Horie Y, Mak TW (2006) Nod1 acts as an intracellular receptor to stimulate chemokine production and neutrophil recruitment in vivo. J Experiment Med 203(1):203–13 10.1084/jem.20051229 PMC2118074 16418393

[236] Tsuji Y, Watanabe T, Kudo M, Arai H, Strober W, Chiba T (2012) Sensing of commensal organisms by the intracellular sensor NOD1 mediates experimental pancreatitis. Immunity 37(2):326–338 22902233 10.1016/j.immuni.2012.05.024 PMC3523885

[237] Gong Z, Lin L, Liu Z, Zhang S, Liu A, Chen L, Liu Q, Deng Y, Xiao W (2019) Immune-modulatory effects and mechanism of action of L-theanine on ETEC-induced immune-stressed mice via nucleotide-binding oligomerization domain-like receptor signaling pathway. J Funct Foods 1(54):32–40

[238] Zhang W, Zhu YH, Yang JC, Yang GY, Zhou D, Wang JF (2015) A selected Lactobacillus rhamnosus strain promotes EGFR-independent Akt activation in an enterotoxigenic Escherichia coli K88-infected IPEC-J2 cell model. PLoS ONE 10(4):e0125717 25915861 10.1371/journal.pone.0125717 PMC4411159

[239] Li XQ, Zhu YH, Zhang HF, Yue Y, Cai ZX, Lu QP, Zhang L, Weng XG, Zhang FJ, Zhou D, Yang JC. Risks associated with high-dose Lactobacillus rhamnosus in an Escherichia coli model of piglet diarrhoea: intestinal microbiota and immune imbalances. 10.1371/journal.pone.0040666 PMC3407149 22848393

[240] Chi H, Chang HY, Sang TK (2018) Neuronal cell death mechanisms in major neurodegenerative diseases. Int J Mol Sci 19(10):3082 30304824 10.3390/ijms19103082 PMC6213751

[241] Fricker M, Tolkovsky AM, Borutaite V, Coleman M, Brown GC (2018) Neuronal cell death. Physiol Rev 98(2):813–880 29488822 10.1152/physrev.00011.2017 PMC5966715

[242] Dygalo NN, Kalinina TS, Bulygina VV, Shishkina GT (2012) Increased expression of the anti-apoptotic protein Bcl-xL in the brain is associated with resilience to stress-induced depression-like behavior. Cell Mol Neurobiol 32:767–776 22278304 10.1007/s10571-011-9794-y PMC11498427

[243] Qin T, Fang F, Song M, Li R, Ma Z, Ma S (2017) Umbelliferone reverses depression-like behavior in chronic unpredictable mild stress-induced rats by attenuating neuronal apoptosis via regulating ROCK/Akt pathway. Behav Brain Res 317:147–156 27646771 10.1016/j.bbr.2016.09.039

[244] Helaly AM, Mokhtar N, Firgany AEDL, Hazem NM, El Morsi E, Ghorab D (2018) Molybdenum bupropion combined neurotoxicity in rats. Regul Toxicol Pharmacol 98:224–230 30081056 10.1016/j.yrtph.2018.08.001

[245] Hou Y, Lou Z, Ji Y, Ruan L, Gao H (2021) Venlafaxine inhibits neuronal apoptosis in a depression rat model via ERK1/ERK2 pathway. Trop J Pharm Res 20(7):1373–1379

[246] Saad MA, El-Sahar AE, Sayed RH, Elbaz EM, Helmy HS, Senousy MA (2019) Venlafaxine mitigates depressive-like behavior in ovariectomized rats by activating the EPO/EPOR/JAK2 signaling pathway and increasing the serum estradiol level. Neurotherapeutics 16:404–415 30361931 10.1007/s13311-018-00680-6 PMC6554373

[247] Song Q, Feng YB, Wang L, Shen J, Li Y, Fan C, Wang P, Yu SY (2019) COX-2 inhibition rescues depression-like behaviors via suppressing glial activation, oxidative stress and neuronal apoptosis in rats. Neuropharmacology 1(160):107779 10.1016/j.neuropharm.2019.107779 31539536

[248] Fan C, Song Q, Wang P, Li Y, Yang M, Yu SY (2018) Neuroprotective effects of ginsenoside-Rg1 against depression-like behaviors via suppressing glial activation, synaptic deficits, and neuronal apoptosis in rats. Front Immunol 9:2889 30581440 10.3389/fimmu.2018.02889 PMC6292928

[249] Rajkowska G, Miguel-Hidalgo J. Gliogenesis and glial pathology in depression. CNS & Neurological Disorders-Drug Targets (Formerly Current Drug Targets-CNS & Neurological Disorders). 2007 Jun 1;6(3):219-33. 10.2174/187152707780619326 PMC2918806 17511618

[250] Serrano A, Haddjeri N, Lacaille JC, Robitaille R (2006) GABAergic network activation of glial cells underlies hippocampal heterosynaptic depression. J Neurosci 26(20):5370–5382 16707789 10.1523/JNEUROSCI.5255-05.2006 PMC6675310

[251] Civita P, Valerio O, Naccarato AG, Gumbleton M, Pilkington GJ (2020) Satellitosis, a crosstalk between neurons, vascular structures and neoplastic cells in brain tumours; early manifestation of invasive behaviour. Cancers 12(12):3720 33322379 10.3390/cancers12123720 PMC7763100

[252] Sugama S (2009) Stress-induced microglial activation may facilitate the progression of neurodegenerative disorders. Med Hypotheses 73(6):1031–1034 19556067 10.1016/j.mehy.2009.02.047

[253] Horikawa H, Kato TA, Mizoguchi Y, Monji A, Seki Y, Ohkuri T, Gotoh L, Yonaha M, Ueda T, Hashioka S, Kanba S (2010) Inhibitory effects of SSRIs on IFN-γ induced microglial activation through the regulation of intracellular calcium. Progress Neuro-Psychopharmacol Biol Psychiat 34(7):1306–16 10.1016/j.pnpbp.2010.07.015 20654672

[254] Zhang Y, Bi X, Adebiyi O, Wang J, Mooshekhian A, Cohen J, Wei Z, Wang F, Li XM (2019) Venlafaxine improves the cognitive impairment and depression-like behaviors in a cuprizone mouse model by alleviating demyelination and neuroinflammation in the brain. Front Pharmacol 5(10):332 10.3389/fphar.2019.00332 PMC6460225 31024304

[255] Zolfaghari SI, Khorasgani MR, Noorbakhshnia M. The effects of lactobacilli (L. rhamnosus, L. reuteri, L. Plantarum) on LPS-induced memory impairment and changes in CaMKII-α and TNF-α genes expression in the hippocampus of rat. Physiology & Behavior. 2021 Feb 1;229:113224. 10.1016/j.physbeh.2020.113224 33127463

[256] Parra I, Martínez I, Vásquez-Celaya L, Gongora-Alfaro JL, Tizabi Y, Mendieta L. Neuroprotective and immunomodulatory effects of probiotics in a rat model of Parkinson’s disease. Neurotoxicity Research. 2023 Apr;41(2):187-200. 10.1007/s12640-022-00627-y 36662412

[257] Zheng G, Wu SP, Hu Y, Smith DE, Wiley JW, Hong S (2013) Corticosterone mediates stress-related increased intestinal permeability in a region-specific manner. Neurogastroenterol Motil 25(2):e127–e139 23336591 10.1111/nmo.12066 PMC3558943

[258] Luo B, Xiang D, Nieman DC, Chen P (2014) The effects of moderate exercise on chronic stress-induced intestinal barrier dysfunction and antimicrobial defense. Brain Behav Immun 39:99–106 24291325 10.1016/j.bbi.2013.11.013

[259] Zong Y, Zhu S, Zhang S, Zheng G, Wiley JW, Hong S (2019) Chronic stress and intestinal permeability: lubiprostone regulates glucocorticoid receptor-mediated changes in colon epithelial tight junction proteins, barrier function, and visceral pain in the rodent and human. Neurogastroenterol Motil 31(2):e13477 30284340 10.1111/nmo.13477 PMC6347514

[260] Fukui H (2016) Increased intestinal permeability and decreased barrier function: does it really influence the risk of inflammation? Inflamm Intest Dis 1(3):135–145 29922669 10.1159/000447252 PMC5988153

[261] McGuinness AJ, Davis JA, Dawson SL, Loughman A, Collier F, O’hely M, Simpson CA, Green J, Marx W, Hair C, Guest G. A systematic review of gut microbiota composition in observational studies of major depressive disorder, bipolar disorder and schizophrenia. Molecular psychiatry. 2022 Apr;27(4):1920-35. 10.1038/s41380-022-01456-3 PMC9126816 35194166

[262] Ding F, Wu J, Liu C, Bian Q, Qiu W, Ma Q, Li X, Long M, Zou X, Chen J (2020) Effect of Xiaoyaosan on colon morphology and intestinal permeability in rats with chronic unpredictable mild stress. Front Pharmacol 16(11):1069 10.3389/fphar.2020.01069 PMC7378849 32765272

[263] Cámara-Lemarroy CR, Guzmán-De La Garza FJ, Cordero-Pérez P, Alarcón-Galván G, Ibarra-Hernández JM, Muñoz-Espinosa LE, Fernández-Garza NE. Bupropion reduces the inflammatory response and intestinal injury due to ischemia-reperfusion. InTransplantation proceedings 2013 Jul 1 (Vol. 45, No. 6, pp. 2502-2505). Elsevier. 10.1016/j.transproceed.2013.04.010 23953570

[264] Charoenphandhu N, Teerapornpuntakit J, Lapmanee S, Krishnamra N, Charoenphandhu J (2012) Duodenal calcium transporter mRNA expression in stressed male rats treated with diazepam, fluoxetine, reboxetine, or venlafaxine. Mol Cell Biochem 369:87–94 22766765 10.1007/s11010-012-1371-2

[265] Mao X, Gu C, Hu H, Tang J, Chen D, Yu B, He J, Yu J, Luo J, Tian G (2016) Dietary Lactobacillus rhamnosus GG supplementation improves the mucosal barrier function in the intestine of weaned piglets challenged by porcine rotavirus. PLoS One 11(1):e0146312 26727003 10.1371/journal.pone.0146312 PMC4699646

[266] Wang Y, Liu Y, Sidhu A, Ma Z, McClain C, Feng W. Lactobacillus rhamnosus GG culture supernatant ameliorates acute alcohol-induced intestinal permeability and liver injury. American Journal of Physiology-Gastrointestinal and Liver Physiology. 2012 Jul 1;303(1):G32-41. 10.1152/ajpgi.00024.2012 PMC3404581 22538402

[267] Forsyth CB, Farhadi A, Jakate SM, Tang Y, Shaikh M, Keshavarzian A (2009) Lactobacillus GG treatment ameliorates alcohol-induced intestinal oxidative stress, gut leakiness, and liver injury in a rat model of alcoholic steatohepatitis. Alcohol 43(2):163–172 19251117 10.1016/j.alcohol.2008.12.009 PMC2675276

